# Metabolic engineering enables* Bacillus licheniformis* to grow on the marine polysaccharide ulvan

**DOI:** 10.1186/s12934-022-01931-0

**Published:** 2022-10-10

**Authors:** Theresa Dutschei, Marie-Katherin Zühlke, Norma Welsch, Tom Eisenack, Maximilian Hilkmann, Joris Krull, Carlo Stühle, Stefan Brott, Alexandra Dürwald, Lukas Reisky, Jan-Hendrik Hehemann, Dörte Becher, Thomas Schweder, Uwe T. Bornscheuer

**Affiliations:** 1 Department of Biotechnology & Enzyme Catalysis, Institute of Biochemistry, University of Greifswald, 17487 Greifswald, Germany; 2 Department of Pharmaceutical Biotechnology, Institute of Pharmacy, University of Greifswald, 17487 Greifswald, Germany; 3 Institute of Marine Biotechnology e.V., 17489 Greifswald, Germany; 4 Max Planck-Institute for Marine Microbiology, 28359 Bremen, Germany; 5 Center for Marine Environmental Sciences (MARUM), University of Bremen, 28359 Bremen, Germany; 6 Department of Microbial Proteomics, Institute for Microbiology, University of Greifswald, 17487 Greifswald, Germany

**Keywords:** Ulvan, Marine polysaccharide, Green algae, Biorefinery process, *Bacillus licheniformis*

## Abstract

**Background:**

Marine algae are responsible for half of the global primary production, converting carbon dioxide into organic compounds like carbohydrates. Particularly in eutrophic waters, they can grow into massive algal blooms. This polysaccharide rich biomass represents a cheap and abundant renewable carbon source. In nature, the diverse group of polysaccharides is decomposed by highly specialized microbial catabolic systems. We elucidated the complete degradation pathway of the green algae-specific polysaccharide ulvan in previous studies using a toolbox of enzymes discovered in the marine flavobacterium *Formosa agariphila* and recombinantly expressed in *Escherichia coli*.

**Results:**

In this study we show that ulvan from algal biomass can be used as feedstock for a biotechnological production strain using recombinantly expressed carbohydrate-active enzymes. We demonstrate that *Bacillus licheniformis* is able to grow on ulvan-derived xylose-containing oligosaccharides. Comparative growth experiments with different ulvan hydrolysates and physiological proteogenomic analyses indicated that analogues of the *F. agariphila* ulvan lyase and an unsaturated β-glucuronylhydrolase are missing in *B. licheniformis*. We reveal that the heterologous expression of these two marine enzymes in *B. licheniformis* enables an efficient conversion of the algal polysaccharide ulvan as carbon and energy source.

**Conclusion:**

Our data demonstrate the physiological capability of the industrially relevant bacterium *B. licheniformis* to grow on ulvan. We present a metabolic engineering strategy to enable ulvan-based biorefinery processes using this bacterial cell factory. With this study, we provide a stepping stone for the development of future bioprocesses with *Bacillus* using the abundant marine renewable carbon source ulvan.

**Supplementary Information:**

The online version contains supplementary material available at 10.1186/s12934-022-01931-0.

## Background

Eutrophication and global warming impact frequency and extent of algal blooming events and thus accumulation of algal biomasses in coastal areas [1–3]. Despite algae or algal products being already used in food, cosmetics, biotechnology and pharmaceutical industry [4–6], washed up algae are still largely unexploited. As a consequence, interest has been raised to develop processes that convert this cheap biomass to valuable products [7] and first attempts are already underway [8]. Amongst a variety of compounds that could be harnessed, polysaccharides are attractive targets. They account for up to 50% of macroalgal biomass and mostly represent cell wall or storage components [9–11]. These polysaccharides are highly diverse in their structure and composition [12]. Targeting this versatile substrate pool thus requires a multitude of enzymes which are usually encoded in highly clustered genomic regions of polysaccharide degrading bacteria. These so-called polysaccharide utilization loci (PUL) encode proteins to mediate binding, degradation and uptake of saccharides [13]. Recently, we were able to elucidate a complex enzymatic cascade to completely deconstruct polymeric ulvan to monomeric sugar compounds using enzymes from the marine flavobacterium *Formosa agariphila* KM3901^T^ recombinantly expressed in *Escherichia coli* [14, 15]. Ulvan is the main cell wall polysaccharide in the green seaweed *Ulva* spp. [16]. The sugar backbone is composed of l-rhamnose, d-xylose and d-glucuronic acid/l-iduronic acid and is highly branched and sulfated. Moreover, the monosaccharide composition varies between species and sampling sites [17]. In *F. agariphila*, ulvan lyases catalyze the initial degradation step, releasing several oligosaccharide species with a 5-dehydro-4-deoxy-d-glucuronate at the non-reducing end [14, 18]. This unsaturated moiety is then removed by glycoside hydrolases (GH), which allows further GH-meditated hydrolysis of oligosaccharides prior to or after their desulfation [14]. On the one hand, such enzyme cascades can be used for the production of rare (sulfated) sugar oligosaccharides that could be interesting due to their immunomodulating activities [19]. On the other hand, these hydrolysates may represent a starting material for biotechnological processes as alternative feedstock for common sugars like glucose for microbial fermentation [5]. Microbial engineering and systems biology can further help to develop such new biomass based bioprocesses [20, 21]. Consequently, in-depth characterization of the selected microbial production species is a prerequisite for strain optimization. The well-established biotechnological work horse *Bacillus licheniformis* is an attractive target to be investigated for the utilization of alternative algal derived biomasses: It produces a variety of enzymes to degrade plant materials, it is a generally recognized as safe (GRAS) strain, has a fast growth rate and is already of high industrial importance [22]. This bacterial cell factory naturally produces the extracellular protease subtilisin [23], which has been developed into industrial production due to its widespread use in detergents [24]. In addition, first processes that use *B. licheniformis* to convert plant biomass into valuable products have already been established. This includes metabolic engineering approaches which enabled the production of acetoin, 2,3-butanediol or lactic acid from kitchen waste or corncob molasses [22, 25–27]. Furthermore, the production of extracellular proteins from algal feedstock [28] was already studied to broaden up the possible use of this bacterium in fermentation processes.

In order to develop an ulvan based bioprocess, we investigated a variety of bacterial strains for their ability to utilize ulvan and identified the industrially relevant bacterium *B. licheniformis* DSM13, which is able to grow on pre-digested ulvan. We investigated strain specific metabolic properties of this bacterium, which are required for ulvan utilization. Our study provides first insights into the development of a potential ulvan based bioprocess with *Bacillus* species.

## Results and discussion

### *Bacillus licheniformis* DSM13 efficiently consumes ulvan-derived monomers

**Fig. 1:**
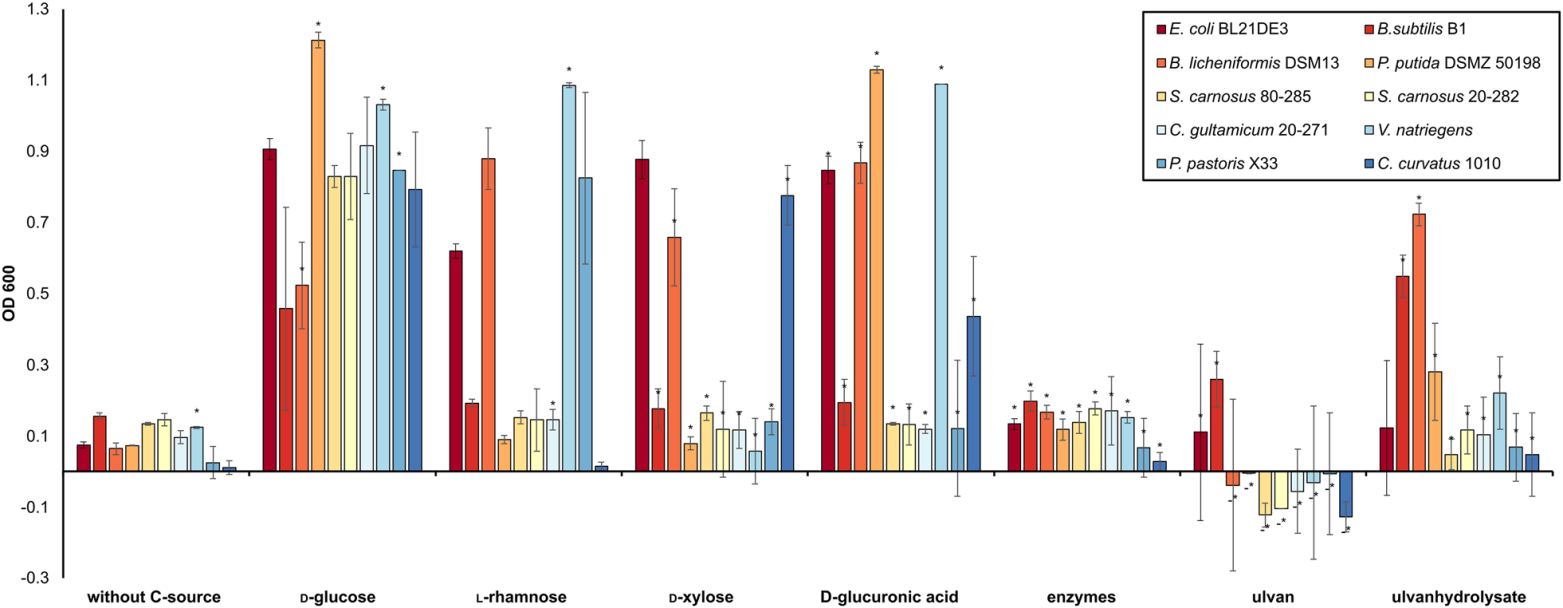
Growth screening of ten different organisms. The growth of these organisms was investigated using ulvan-derived monosaccharides (rhamnose, xylose, glucuronic acid), ulvan and ulvan hydrolysate B (UHB) which was generated with *F. agariphila* enzymes recombinantly expressed in *E. coli* (see Additional file 1: Table S1, Reisky et al. [14]). Cultures were grown in triplicates (*duplicates) in 96 deep-well plates in 1 mL culture volume. The OD600nm was measured after 48 h

In a first attempt, we screened 10 different strains for their ability to grow on ulvan and ulvan-derived monosaccharides, as single monomers or as monosaccharide mixture (Fig. 1, Additional file 1: Fig. S1). While none of the strains grew on raw ulvan, *B. licheniformis* DSM13, *Cryptococcus curvatus* 1010 and *Pseudomonas putida* DSMZ 50198 consumed the monomer cocktail derived from ulvan digestion using the complete enzymatic cascade of *F. agariphila* that was recombinantly expressed in *E. coli* BL21(DE3) as described previously (Additional file 1: Table S1, Fig. S2) [14, 15].

This mixture, ulvan hydrolysate B (UHB), provided l-rhamnose, d-xylose, d-glucuronic acid and 5-dehydro-4-deoxy-d-glucuronate. *B. licheniformis* DSM13 grew also well on each individual monosaccharide present in the mixture, as described before [29, 30], even better than on d-glucose. Although *P. putida* DSMZ 50198 and *Bacillus subtilis* B1 consumed UHB, they were not able to grow on l-rhamnose (Fig. 1), which is known for *P. putida* [31], but disagrees with observations reported for *C. curvatus* 1010 [32]. *P. putida* DSMZ 50198 also lacks the ability to grow on d-xylose. Growth experiments identified *B. licheniformis* DSM13 as a suitable candidate for further investigations to establish an ulvan sugar-based bioprocess.

### *Bacillus licheniformis* DSM13 grows and accumulates proteases on fully digested ulvan

**Fig. 2:**
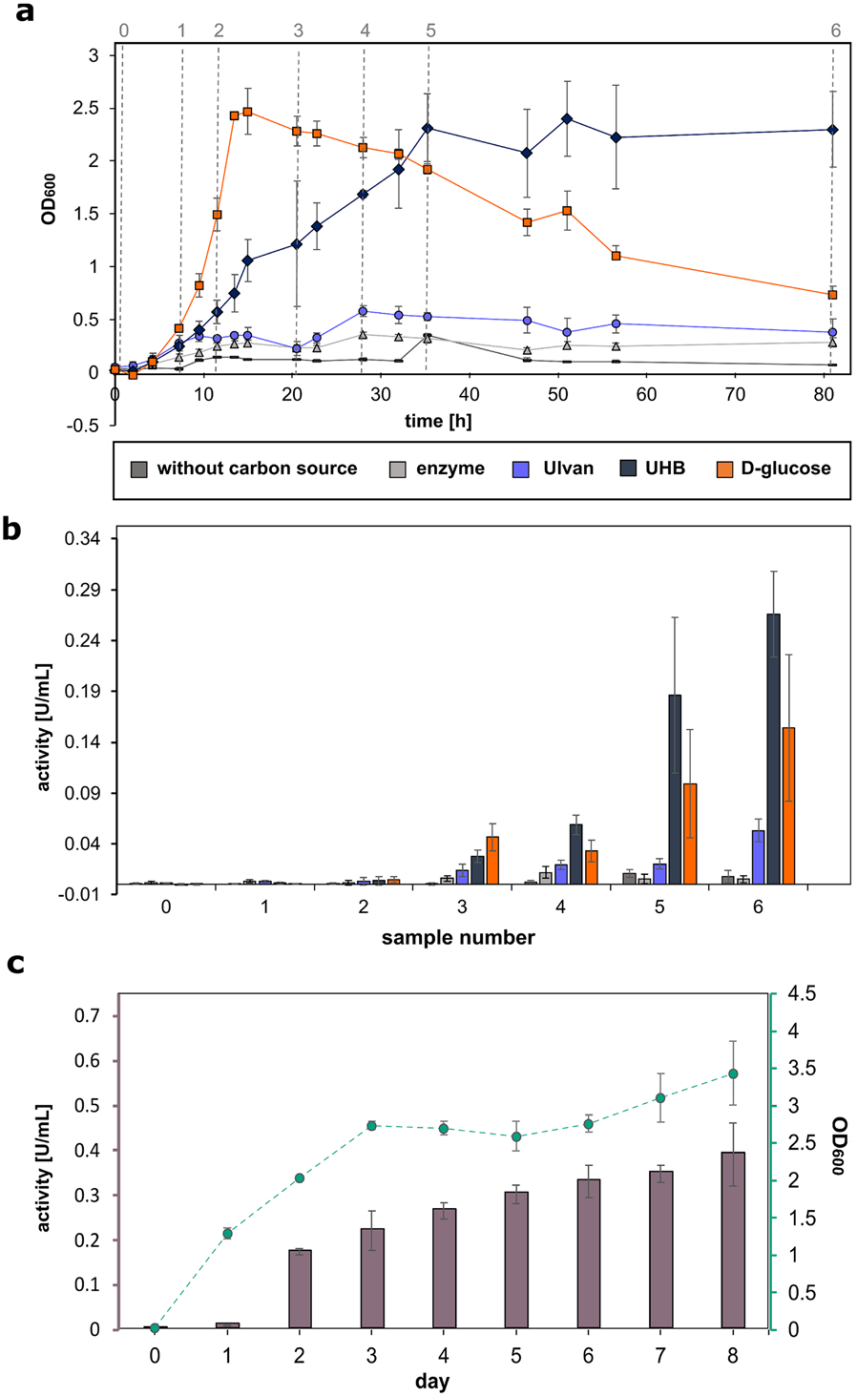
Protease production of *B. licheniformis* DSM13 during growth on different substrates. **a** *Bacillus licheniformis* was grown in M9-mineral salts medium supplemented with ulvan, ulvan hydrolysate (UHB) (Additional file 1: Tables S1, S3 and S4) or glucose for 80 h. Cultures without added carbon source and with or without the ulvan hydrolysing enzymes (see Additional file 1: Table S1) served as controls. Dotted lines mark time points for **b** determination of protease activity using the AAPF test. **c** Growth on UHB over 8 days with corresponding protease activity measurements

To investigate the suitability of the abundant macroalgal polysaccharide ulvan as feedstock for production processes, we quantified exemplarily protease activity via the AAPF-assay [33] during cultivation, like the alkaline serine protease (AprX, Q65IP4), subtilisin protease Apr (Q65LP7) and extracellular serine protease Vpr (Q65DN2). Following growth over time, *B. licheniformis* DSM13 grew more slowly on UHB compared to d-glucose, but reached a comparable maximum optical density that was stable until the end of the experiment (Fig. 2a). At the same time, protease activity increased over time (Fig. 2b) and was stable even in prolonged cultivations (Fig. 2c). These growth experiments revealed a constant stationary phase over more than 5 days for *B. licheniformis* using UHB as the sole carbon source. The resulting increased biomass until the end of the cultivation improved protease production significantly compared to the glucose-based cultivations.

### Capabilities of *B. licheniformis* DSM13 to grow on ulvan-derived oligosaccharides

**Fig. 3:**
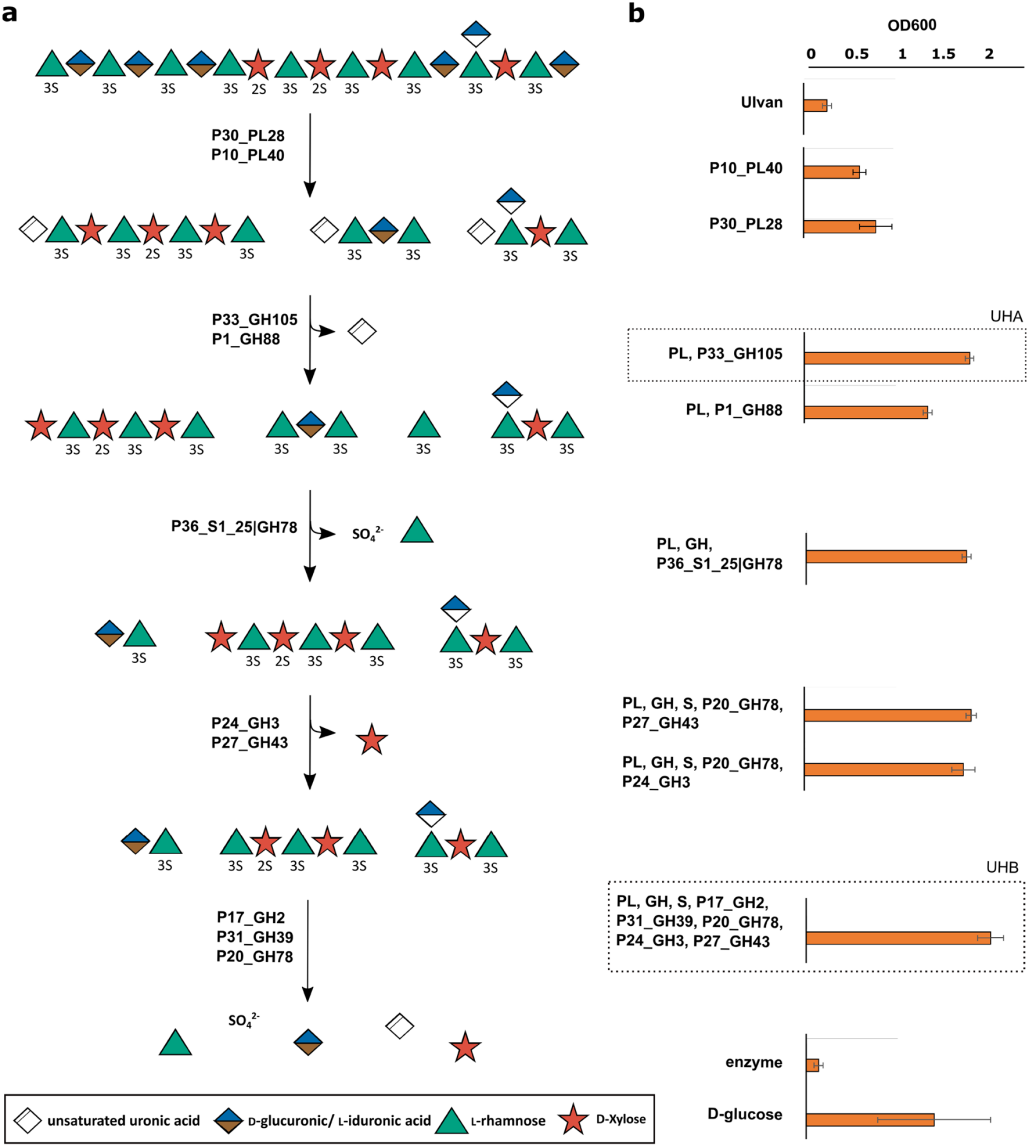
In-depth analysis of the capability of *B. licheniformis* DSM13 to degrade ulvan-derived oligosaccharides. Ulvan was digested with selected enzymes or enzyme cocktails, described before [14, 15], to produce a total of 12 different ulvan hydrolysates (Additional file 1: Fig. S4). **a** These vary in their mono- and oligosaccharide content based on **b** the enzymes used and thus provide specific carbon sources for *B. licheniformis* DSM13. The DSM13 strain was cultivated in M9-mineral medium supplemented with ulvan or enzyme-generated ulvan hydrolysates and OD600 was measured after 24 h. Growth on hydrolysates UHA and UHB, which were used for further investigations, is highlighted. (PL: mix of P10_PL40; GH: mix of P33_GH105, P1_GH88; S: P36_S1_25|GH78)

The initial growth experiments demonstrated the physiological capability of *B. licheniformis* DSM13 to utilize ulvan-specific monosaccharides (Fig. 2, Additional file 1: Fig. S3). Consumption of these monosaccharides as well as the fact that this bacterium is well known to degrade plant material [34, 35], gave reasons to suspect also the acceptance of ulvan-derived oligosaccharides. Therefore, 12 different ulvan hydrolysates were examined as potential substrates (Additional file 1: Fig. S4). These enzymatically digested ulvan-extracts cover different levels of ulvan depolymerisation as described in our previous studies and thus differ in their mono- and oligosaccharide composition [14, 15]. Again, each hydrolysate, including the aforementioned UHB, was produced using selected *F. agariphila* ulvan-degrading enzymes recombinantly expressed in *E. coli* BL21(DE3) (Additional file 1: Table S1, Fig. S2; Fig. 3a) [14]. The cell densities achieved after 24 h of cultivation identified the required level of hydrolyzation to allow growth of *B. licheniformis* DSM13. At the same time, they indicated which enzymatic activities might be missing in *Bacillus* and would thus enable growth on higher degrees of polymerization or ulvan itself (Fig. 3b). The ulvan lyase-generated hydrolysates improved digestibility only to a small extent (P30_PL28 > P10_PL40), similar to P31_GH39 and P17_GH2 pre-digestion (Additional file 1: Fig. S4).

However, optical densities were considerably increased if the ulvan lyase activity of P30_PL28 was either supported by the unsaturated glucuronyl hydrolase P1_GH88 or the glycoside hydrolase P33_GH105 (UHA). This may be due to the release of smaller oligosaccharides and unsaturated uronic acids as carbon source in UHA. The P30_PL28 ulvan lyase cleaves the ulvan polymer between α-l-rhamnose-3-sulfate-(1,4)-β-d-glucuronic acid, which produces an unsaturated uronic acid at the non-reducing end of the released oligosaccharide, which is specific for lyases. This unsaturated uronic acid (4-deoxy-α-l-threo-hex-4-enopyranuronic acid) is then hydrolyzed by P33_GH105. Indeed, previous growth experiments confirmed *B. licheniformis* DSM13 to consume 4-deoxy-α-l-threo-hex-4-enopyranuronic acid (Additional file 1: Fig. S3). This way, not only easily digestible monosaccharides are released from oligosaccharides using P33_GH105, its activity also enables P30_PL28 to cleave the oligomer even further since lyase products inhibit subsequent lyase activities [14, 18]. In addition, unsaturated uronic acids in oligosaccharides might hinder their subsequent disassembling by *B. licheniformis* DSM13.

Interestingly, additional hydrolysis steps, which also included sulfatases, did not further improve growth. This led to the assumption that the two initial major enzyme activities of the ulvan degradation pathway [14], ensured by the ulvan lyase (PL28) and unsaturated glucuronyle hydrolases (GH105, GH88), provided an oligosaccharide mixture suitable for *B. licheniformis* DSM13 to degrade ulvan. This also indicated the availability of putative CAZymes in *B. licheniformis* DSM13 to utilize l-rhamnose, d-xylose and d-glucuronic acid from ulvan oligomers and to channel them into its carbon and energy metabolism.

### Proteogenomic analysis of *B. licheniformis* DSM13

To further interpret our results and to explore the physiological potential of *B. licheniformis* to utilize ulvan derived sugars, we performed computational and proteome analyses. We analyzed the intracellular soluble as well as the extracellular proteomes of ulvan-, UHA- and UHB-cultivated cells (Fig. 3, Additional file 1: Fig. S5) compared to rhamnose and glucose cultures. In general, it is well known that *B. licheniformis* DSM13 secretes a variety of extracellular CAZymes to degrade polysaccharides [34, 35]. Correspondingly, computational analysis with the web server for automated CAZyme annotation, dbCAN2 [36, 37], identified 86 PLs, GHs and CEs to be encoded in its genome, 58 of which were captured by intracellular and extracellular proteomes (Additional file 2: Table S6; Additional file 1: Fig. S5).

### Enzymes to cleave ulvan are lacking

Proteome and dbCAN2 analyses did not reveal suitable ulvanolytic enzyme activities of *B. licheniformis* DSM13 wild type strain, which are required for the initial digestion of ulvan, and thus confirmed our growth experiments shown in Fig. 2. The strain lacks PLs from families 24, 25, 28 and 40 [12] to cleave ulvan into oligosaccharides. Moreover, proteome analyses do not indicate PLs or GHs that depolymerize pectin and pectin components to also cleave ulvan, since corresponding proteins were either low abundant (PL11_1 Q65KY4) or quantified across all samples (PL1_5 Q65DC2, PL3_1 Q65EF5 and GH28 Q65F26, Additional file 1: Fig. S5; Additional file 2: Table S6). *B. licheniformis* DSM13 encodes two GH105s (Q65FY9, Q65KY9) as candidates to catalyze the next necessary enzymatic step in ulvan disassembling, but both of them were not detected in our proteome analyses during growth on ulvan oligosaccharides. Instead, they might be involved in rhamnogalacturonan I degradation, like in *B. subtilis* [39].

### GHs that may disassemble oligosaccharides

**Fig. 4:**
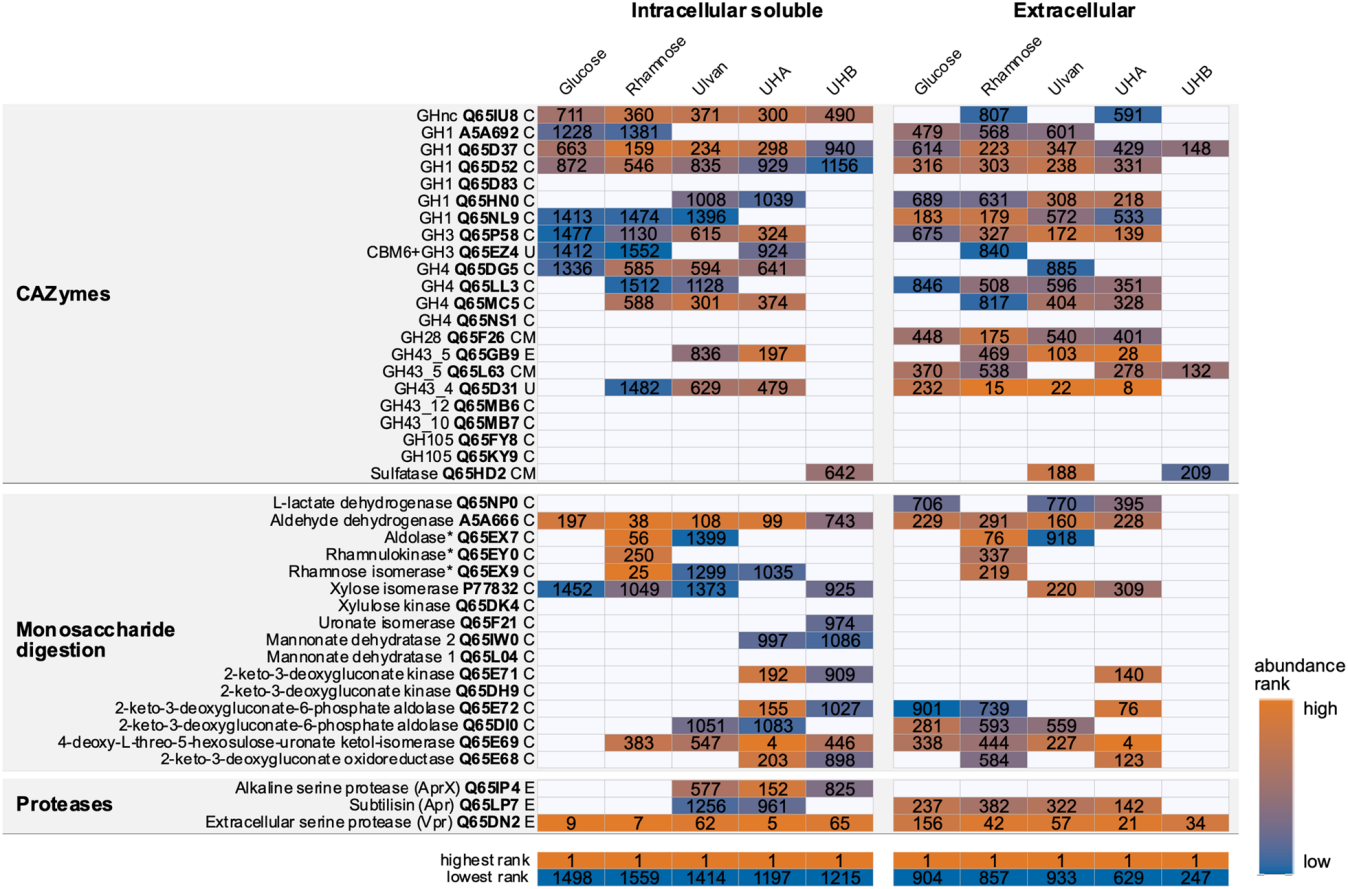
*B. licheniformis* DSM13-encoded proteins that may contribute to ulvan-derived oligo- and monosaccharide degradation and their abundance in the intracellular soluble and extracellular proteomes. The graph highlights the relative abundance of proteins within the respective sample given as abundance ranks. Abundance ranks were derived from %riBAQ values (Additional file 1: Table S6). The lowest rank corresponds to the total number of quantified proteins per sample. Blank tiles represent proteins that were not quantified. Note that the enrichment of protein fractions is not exclusive and overlaps occur, e.g., due to cell lysis or intracellular production of extracellular enzymes. Function, protein ID and suggested localization (PSORTb v3.0.2) [38] are indicated. *C* cytoplasmic, *CM* cytoplasmic membrane, *E* extracellular, *U* unknown, *UHA/B* ulvan hydrolysate A/B (Fig. 3a). *Adapted based on BlastP searches, Q65EX7 formerly annotated as putative oxidoreductase YuxG, Q65EY0 as putative carbohydrate kinase YulC, Q65EX9 as putative xylose isomerase

Nevertheless, the adaptation of *B. licheniformis* DSM13 to pectin or hemicellulose usage may still allow for consumption of certain ulvan oligosaccharides as demonstrated by our growth experiments. Proteome analyses captured potentially involved GH43s, the GH43_4 (Q65D31) being highly abundant in ulvan and ulvan hydrolysate samples (1–3% of the total extracellular proteome) (Fig. 4, Additional file 2: Table S6, S7). GH43_4 (Q65D31, YxiA/Abn2) as well as GH43_5 (Q65GB9, AbnA) are both extracellular enzymes that degrade arabinans [40, 41]. By contrast, in *F. agariphila* a GH43_10 cleaved xylose moieties from ulvan-derived oligosaccharides [14]. The corresponding *B. licheniformis* DSM13 enzyme (Q65MB7) was not quantified by proteome analyses. However, several other GHs of family 1, 3 and 4 that might have xylosidase activity, as well as an unclassified (nc) GH, were quantified in the proteome of *B. licheniformis* DSM13 grown on ulvan extracts.

### The involvement of sulfatases remains speculative

Ulvan degradation does not only require PLs and GHs to cleave the sugar chain, but also sulfatases to act on sulfated rhamnose or xylose units. To encounter the complexity of ulvan composition and its degree of sulfation, marine ulvan targeting strains encode a set of sulfatases [14, 42–44], e.g., eight sulfatases from five S1 subfamilies are encoded in the *F. agariphila* ulvan PUL. In *B. licheniformis*, only three proteins are annotated as putative sulfatases. However, for two of them, YfnI (Q65D92) and YflE (Q62XX8), it has been discovered that they are involved in cell wall lipoteichoic acid synthesis in *B. subtilis* (Additional file 1: Fig. S6) [45]. Indeed, the remaining sulfatase (Q65HD2) was abundant in ulvan and UHB secretomes (Fig. 4). We therefore cloned and overexpressed the respective gene in *E. coli*, but so far, no specific sulfatase activity of this enzyme could be detected (data not shown). At the same time, since UHB hydrolysate provides desulfated monosaccharides, its role in desulfation needs to be investigated in more detail in future studies. Nevertheless, the results underline that sulfatases are largely underexplored in *B. licheniformis* and might not even be recognized as such, e.g. alkaline phosphatases preferentially cleave phosphate monoesters, but are also active on the sulfate counterparts [46]. In another scenario, *B. licheniformis* DSM13 could just consume desulfated ulvan fragments.

### Consumption of ulvan-derived monosaccharides

In case of UHA the hydrolysate does not only contain oligosaccharides, but also free unsaturated uronic acids as substrates, which was demonstrated to be consumed by our growth experiments (Additional file 1: Fig. S3). Confirming this, a 4-deoxy-L-threo-5-hexosulose-uronate ketol-isomerase (Q65E69) was among the most abundant proteins in UHA samples (Fig. 4) representing 1.6% and 4.5% of the total UHA intracellular and extracellular proteome, respectively (Additoinal file 2: Table S6, Additional file 3: Table S7). Pathways for the other monosaccharides could also be mapped in UHA and UHB samples (Fig. 4), although they were not fully covered. Taking multiple samples over time and comparing them to respective monosaccharide cultures could close these gaps. Monosaccharides are probably consumed successively, as glucose and xylose are not degraded simultaneously in *Bacillus* species [47, 48].

### Protease expression during growth on ulvan and oligosaccharides

In addition, the detected significantly increased protease activities of *B. licheniformis* cultivations with ulvan hydrolysates (see Fig. 2) indicated an elevated protease expression under these conditions. This was supported by our proteome analyses, where the alkaline serine protease (AprX, Q65IP4) was only quantified in ulvan and ulvan hydrolysate samples. However, the subtilisin protease Apr (Q65LP7) or the extracellular serine protease Vpr (Q65DN2) were present throughout all conditions, with high levels of Vpr (Fig. 4). Whereas putative algal-derived proteins from extraction were probably negligible (ulvan, UHA and UHB samples) as inducers of these enzymatic activities, the added *F. agariphila* enzyme extracts to generate ulvan hydrolysates injected additional protein sources into our samples. However, it is worth emphasizing that this potential nutrient source did not cause a significant biomass increase in our control growth experiments (Fig. 2a, enzyme control). The observed increased protease activities thus underline the suitability of ulvan and hydrolysates thereof as potential substrates for industrial bulk protease production processes.

### Functional expression of two initial ulvan-degrading CAZymes in *Bacillus*

**Fig. 5:**
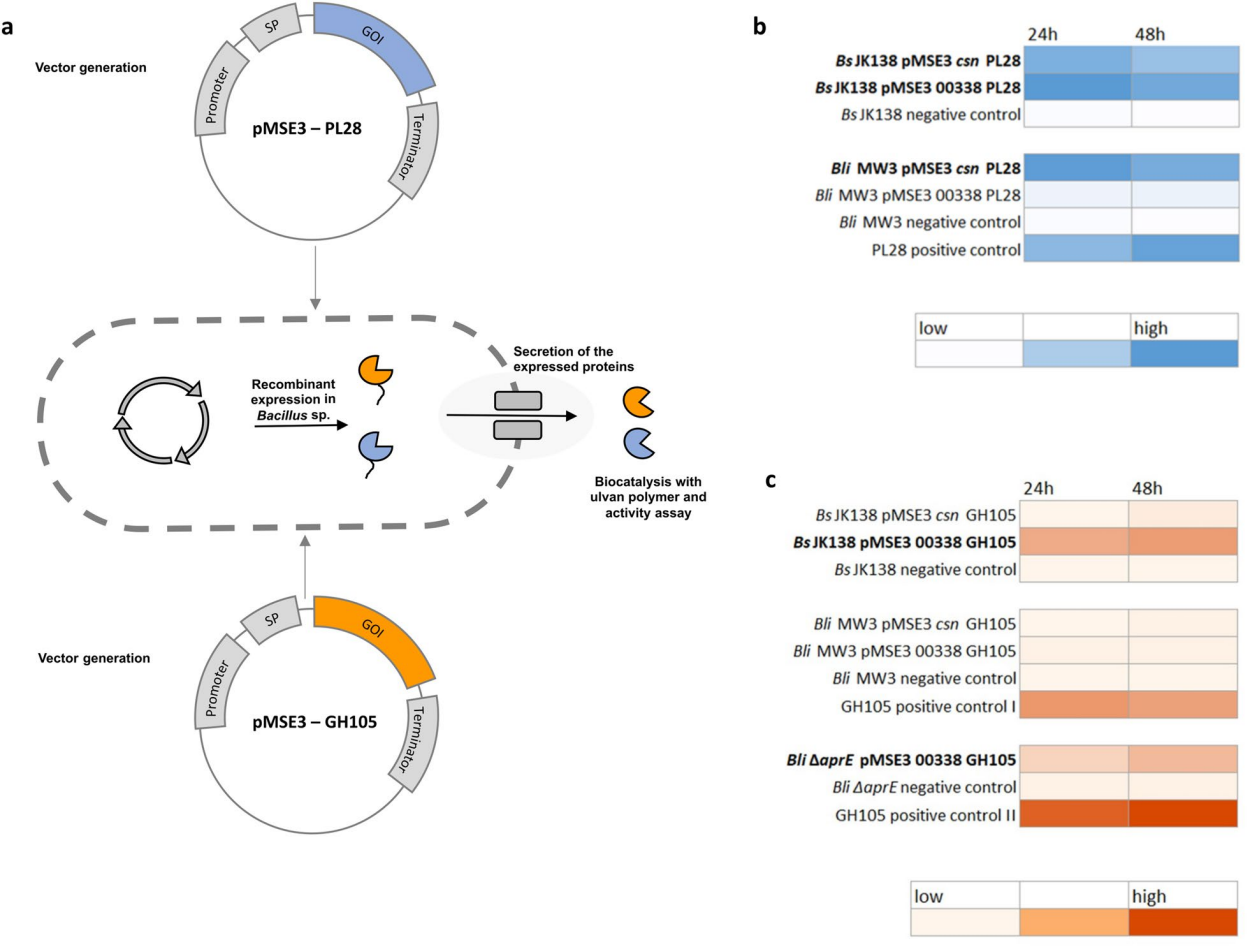
Integration of two genes of ulvan-degrading CAZymes in *Bacillus* strains. High copy expression vectors for synthesis of PL28 and GH105 enzymes were constructed, integrated and functionally expressed in *Bacilli* (**a**). Extracellular PL28 (**b**) and GH105 (**c**) activities detected by lyase-assay and thiobarbituric acid-assay, respectively (Additional file 1: Figs. S8–S10)

Our previous experiments have shown that *B. licheniformis* DSM13 lacks two initial enzyme activities, ulvan lyase (PL28) and unsaturated glucuronyl hydrolase (GH105, GH88), to use the ulvan polymer as sole carbon source. Therefore, we integrated the *F. agariphila* P30_PL28 and P33_GH105 into a *Bacillus* host-vector system, expressing them as secreted proteins to disassemble ulvan and thus enabling a self-sufficient *Bacillus* strain (Fig. 5a). Since *B. subtilis* and *B. licheniformis* share a similar CAZyme repertoire [35, 49] and showed similar growth in comparative experiments (Additional file 1: Fig. S7), *B. subtilis* JK138 and *B. licheniformis* MW3 were selected as first expression hosts. *B. licheniformis* MW3, a derivative of *B. licheniformis* DSM13, lacks the RM-system (restriction and modification system) which facilitates the genetic accessibility of this strain [50]. Starting with the signal peptide *csn* from *B. subtilis* and *00338* from *B. licheniformis* for both hosts, proteins were expressed in an active form extra- and intracellularly after growth in EnpressoB under simulated fed-batch conditions (Additional file 1: Fig. S9). PL28 synthesis and activity was confirmed for both expression hosts by ulvan lyase assays (Fig. 5b) and C-PAGE analysis (Additional file 1: Figs. S9, S10).

Production of the GH105 enzyme was detected in *B. subtilis* in combination with the 00338-secretion signal but not in *B. licheniformis* MW3, which was most probably caused by protease activity in this strain. Thus, we additionally used another *B. licheniformis* expression strain (*B. licheniformis* SH006), which is similar to *B. subtilis* JK138 being deficient in the main extracellular protease Apr. Indeed, functional expression of GH105 was detected in the *B. licheniformis* protease-mutant strain using the same expression cassette as for *B. subtilis* (Fig. 5b). Corresponding carbohydrate polyacrylamide gel electrophoresis (C-PAGE) analysis for GH105 activity is shown in Additional file 1: Fig. S10. Although enzyme activity was higher within the extracellular fraction, intracellular enzyme activities for PL28 and GH105 were measured in the activity assays (data not shown) and were also detected by C-PAGE (Additional file 1: Fig. S10), indicating an incomplete protein secretion. To further improve protein secretion of PL28 and GH105, all Sec dependent signal peptides of *B. subtilis* were screened to enhance protein secretion. For this purpose, the *B. subtilis* Secretory Protein Expression System (Takara Clontech) was used, which allows the fusion of 173 Sec-dependent signal peptides of *B. subtilis* to the genes of interest. Based on *B. subtilis* JK138, it could be demonstrated, that higher enzyme activities for PL28 were measured when protein secretion was mediated by the secretion signal of *wprA*, whereas none of the investigated signal peptides mediated an increase in GH105 activity (data not shown). Taken together, we were able to establish the functional expression of the two initial ulvan degrading enzymes PL28 and GH105 in *B. subtilis* and *B. licheniformis,* which may enable both organisms for applications in bioprocess development based on the alternative biomass ulvan.

### Co-expression of the PL28 and GH105 in *B. licheniformis* SH006

**Fig. 6:**
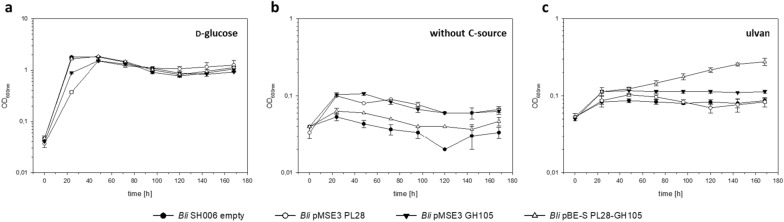
Growth of the *B. licheniformis* expression strains on ulvan. *B. licheniformis* SH006 (black circles), *B. licheniformis* pMSE3 PL28 (white circles), *B. licheniformis* pMSE3 GH105 (black triangles) and co-expression strain *B. licheniformis* pBE-S PL28-GH105 (white triangles) were grown in M9 medium supplemented with glucose (**a**), M9 without carbon source (**b**) and M9 supplemented with ulvan (**c**) at 30 °C and 250 rpm (for 1 week)

As soon as the functional expression of either the PL28 or the GH105 encoding gene was established, a self-sufficient strain was designed by combining both enzyme genes. In order to compare PL28 or GH105 single expression vs. co-expression of both marine enzymes in *B. licheniformis* SH006, growth experiments for protein expression (Additional file 1: Fig. S11) and ulvan utilization (Fig. 6) were carried out simultaneously for the *B. licheniformis* “empty” strain, serving as the negative control, for *B. licheniformis* pMSE3 P_*aprE*_* csn*-UL, *B. licheniformis* pMSE3 P_*aprE*_ 00,338-GH and *B. licheniformis* pBE-S PL28-GH105. Determination of enzymatic activities revealed functional expression of the PL28 enzyme in the PL28 single expression strain *B. licheniformis* pMSE3 PL28 and the co-expression strain *B. licheniformis* pBE-S PL28-GH105 (Additional file 1: Fig. S12a) whereas GH105 activity was detected in the *B. licheniformis* pMSE3 GH105 and *B. licheniformis* pBE-S PL28-GH105 strains for both investigated time points (Additional file 1: Fig. S12b). As illustrated in Additional file 1: Fig. S12a, the measured PL28 activities in *B. licheniformis* pBE-S PL28-GH105 were very low after 24 h and even in a negative range after 48 h of expression. However, this represents a strong hint for co-expression of both enzymes: the unsaturated uronic acid formed by the PL28 led to an increased absorption (A_235nm_), which was then reversed by the GH105 that cleaved this moiety. Corresponding C-PAGE analysis for PL28 and GH105 showed activities in all analyzed strains as shown in Additional file 1: Fig. S13. Taken together, the data of our protein expression experiments under simulated fed-batch conditions in EnpressoB medium clearly demonstrated that both marine enzymes were actively co-expressed.

In a final experiment, we thus wished to demonstrate, that *B. licheniformis*, equipped with the pBE-S PL28-GH105 co-expression vector, is able to grow on ulvan as the sole carbon source. Therefore, the same four expression strains were grown for 7 days in: (i) M9 mineral medium without carbon source, serving as the negative control; (ii) M9 mineral medium with 0.4% d-glucose, serving as the positive control; and (iii) with 1% ulvan as the sole carbon source (Fig. 6). While the single PL28 and GH105 strains lack the ability to grow on the ulvan, the co-expression strain shows an increased growth over this period of time. A C-PAGE of the culture’s supernatant showed ulvan hydrolysis (Additional file 1: Fig. S14) in the PL28 and co-expression cultivation. This demonstrated PL28 and GH105 expression and activity in the M9-mineral medium supplemented with ulvan. Additionally, this experiment confirmed that the *B. licheniformis* strain needs PL28 and GH105 to grow on ulvan biomass.

## Conclusion

This study reveals the promising metabolic potential of the bacterial cell factory *B. licheniformis* to utilize the abundant and renewable marine algal polysaccharide ulvan. We demonstrated that the native *B. licheniformis* DSM13 strain can grow on ulvan-derived oligo- und monosaccharides obtained by enzymatic pre-hydrolysis. Our proteogenomic analyses indicated that *B. licheniformis* DSM13 lacks the initial ulvan degradation enzymes, but that the pre-digestion of this marine polysaccharide with two particular enzymes suffices to generate a suitable carbon source for this bacterium. We demonstrate that a metabolically engineered *B. licheniformis* strain, equipped with two marine heterologously expressed marine enzymes for the initial breakdown of the algal polysaccharide ulvan, is able to grow on ulvan as the sole carbon and energy source. Thus, this study pinpoints a suitable metabolic engineering strategy for future strain development aiming for a cell factory for the conversion of the abundant marine polysaccharide ulvan as alternative feedstock in large-scale bioprocesses.

## Methods

### Genes and enzyme expression

We used the already available pET28a(+) based expression constructs, coding for the *Formosa agariphila* KMM3901^T^ (collection number DSM15362 at DSMZ, Braunschweig, Germany) specific ulvan enzyme cascade [14]*.* Enzyme overproduction in *E. coli* was performed as described previously [14]*.* After cell lysis the soluble fraction was filtered (0.45 µm) and the resulting crude extract was aliquoted and shock frozen. The enzyme expression was confirmed via SDS-PAGE (Additional file 1: Fig. S2).

### Extraction of ulvan

Dried *Ulva* biomass from the Atlantic coast in Spain was purchased as organic sea lettuce (Kulau, Berlin, Germany). Ulvan was extracted as described before, but distilled water was used as extraction solvent [14].

### Enzyme assays

The thiobarbituric acid assay [51] adapted for reduced volumes detected 5-dehydro-4-deoxy-d-glucuronate in the culture supernatant. The protease/peptidase activity in the culture supernatant was determined via the AAPF-assay, through the release of *p-*nitroanilin (410 nm, E^M^ = 9800) from the substrate *N*-*succinyl*-Ala-Ala-Pro-Phe-*para*-nitroanilide (succinyl-AAPF-*p-*Nitroanilide). The enzyme activity was calculated from the amount of *p-*nitroanilin released per time [33].

### Strains

Ten microorganisms were selected for growth screening on ulvan extracts or hydrolysate: *Escherichia coli* Top10*, E. coli* BL21(DE3), *Bacillus subtilis* B1, *Saccharomyces cerevisiae* GRF18, *Vibrio natriegens* ATCC 14048, *Pseudomonas putida* DSMZ 50198, *Pichia pastoris* X33, *Bacillus licheniformis* DSM13, *Cupriavidus necator* H16 and *Cutaneotrichosporan curvatus* DSM 101032 (Additional file 1: Table S2). All strains were maintained as glycerol stocks, stored at − 80 °C. The Bacilli strains *Bacillus subtilis* JK138, *Bacillus licheniformis* MW3 and *Bacillus licheniformis* SH006 were used for all expression experiments in this study. *E. coli* DH10B (Invitrogen, Darmstadt, Germany) [F-endA1 recA1 galE15 galK16 nupG rpsL ΔlacX74 Φ80lacZΔM15 araD139 Δ(ara,leu)7697 mcrA Δ(mrr-hsdRMS-mcrBC) λ-] was used as the host strain for all subcloning procedures. *Bacillus licheniformis* DSM13 mutant SH006 was constructed with a homologous recombination method using a pE194-derived shuttle vector pE194SV analogous to the pMAD system [52]. pE194SV consist of the temperature-sensitive ori and erythromycin resistance marker gene from pE194ts [53] cloned into the *Sma*I site of pUC18, in which the native *Bsa*I site was removed. Moreover, pE194SV carries a type-II-assembly mRFP cassette from pBSd141R [54]; GenBank accession number: KY995200) integrated into the *Bam*HI site of pUC18. The pE194SV based gene deletion procedure was conducted according to Nahrstedt et al*.* 2005, using 45 °C instead of 42 °C as non-permissive temperature [55].

For the deletion of the restriction endonuclease (*hsdR1*) within the restriction modification operon 1 and the adjacent *mcrA* gene, 5′- and 3′-homologous flanking regions were PCR amplified from DSM13 genomic DNA. The 5′-flanking region was amplified using primers P1-hsdR1 and P2-hsdR1 and the 3′-flanking region was amplified with primers P3-hsdR1 and P4-hsdR1. Primers P1-hsdR1 and P4-hsdR1 introduced *Bsa*I cut sites and unique overhangs for subsequent cloning. Both fragments were ligated by SOE-PCR [56] and cloned via *Bsa*I into pE194SV, resulting in plasmid pDhsdR1.

For the deletion of the restriction endonuclease (*hsdR2*) within the restriction modification operon 2, 5′- and 3′-homologous flanking regions were PCR amplified from DSM13 genomic DNA. The 5′-flanking region was amplified using primers P1-hsdR2 and P2-hsdR2 and the 3′ flanking region was amplified with primers P3-hsdR2 and P4-hsdR2. Primers P1-hsdR2 and P4-hsdR2 introduced *BsmB*I cut sites and unique overhangs for subsequent cloning. Both fragments were then ligated by SOE-PCR, digested with *BsmB*I and cloned into the *Bsa*I digested pE194SV, resulting in plasmid pDhsdR2.

For the deletion of the poly-γ-glutamic acid (*pga*) synthesis operon (*pgsBCAE*) and the *apr* gene encoding an extracellular alkaline serine protease individual cassettes comprising the 5′- and 3′-homologous flanking regions, flanked by *Bsa*I cut sites and unique overhangs were ordered as synthetic fragments. Each cassette was cloned separately via *Bsa*I into pE194SV, resulting in plasmids pDpga (∆*pga*) and pDapr (∆*apr*).

### Preparation of a sugar rich hydrolysate

2 mg/mL ulvan in phosphate buffer (25 mM, 50 mM NaCl, pH 7.5) was incubated with 0.5% (v/v) of the respective *F. agariphila* crude enzyme (Additional file 1: Table S1) overnight. The ulvan hydrolysates were centrifuged for 5 min at 4500x*g* and were then filtered (0.2 µm).

### Monosaccharide composition analysis of the cultivation media

The ulvan, UHA and UHB raw media (Additional file 1: Fig. S1) were chemically hydrolysed (1 M HCl for 24 h at 100 °C). Afterwards, the samples were filtered (0.2 µm Spin-X filter) prior to HPAEC-PAD analyses using a Dionex CarboPac PA10 column (Thermo Fisher Scientific, Waltham, Massachusetts, USA) and monosaccharide mixtures as standards for column calibration [57].

### Cultivation of different strains with various carbon sources

The M9-mineral medium with 0.2% (w/v) yeast extract was supplemented with various sugar sources (Additional file 1: Tables S3, S4). Selected monosaccharides were d-glucose, l-rhamnose, d-xylose and d-glucuronic acid, each at a final concentration of 0.4%(w/v). A final concentration of 1%(w/v) was used in case of ulvan and ulvan hydrolysates. M9 and M9 supplemented with enzyme mixtures from the preparation of the sugar rich hydrolysates were used as controls. Precultures were prepared in respective rich media for the corresponding microorganisms (LB media, YPD for yeasts) and overnight (for yeasts 1.5 days). This preculture was used to inoculate a second preculture in M9-mineral media with 0.2%(g/L) glucose as carbon source (1:100). The main culture was inoculated (1:100) with the M9-mineral media preculture and cultured up to 4 days at 30 °C and 180 rpm. The optical density was measured at 600 nm.

### Proteome analyses

For proteome analyses, late logarithmic phase cells from triplicates of ulvan, UHA, UHB, l-rhamnose and d-glucose cultures were separated from supernatants by centrifugation (20 min, 4000×*g*, 4 °C). Intracellular soluble proteins were extracted by suspending cell pellets in lysis buffer (4% SDS, 1% NaDCA, 50 mM TEAB) adapted to Hinzke et al*.* [58]. Samples were incubated for 5 min at 600 rpm and 95 °C, then cooled on ice shortly and sonicated for 5 min. Cell debris was removed from the protein extract (intracellular soluble proteome) by centrifugation (10 min, 14,000×*g*, room temperature). Protein concentration was determined using the Pierce™ BCA Protein Assay Kit (Thermo Fisher Scientific, Waltham, Massachusetts, US). Secreted and detached proteins (extracellular proteome) were extracted from cultivation supernatants using StrataClean beads (Agilent, Santa Clara, California, US) [59]. In brief, 20 µL of bead solution, extracting approximately 20 – 30 µg of protein, was removed. Beads were primed in 180 µL 37% hydrochloric acid (100 °C, 6 h) and then washed in TE buffer (50 mM Tris, 10 mM EDTA, pH 8.0) twice (5 min, 3500×*g*, room temperature). 0.2 µm-filtered supernatants were incubated with prepared beads overnight in a 360° rotating shaker at 8 rpm and 4 °C. The protein-loaded beads were pelleted by centrifugation (45 min, 10,000×*g*, 4 °C) and washed in TE buffer. In a last step, they were resuspended in 1 mL of ultrapure water and dried by vacuum centrifugation. 25 µg of protein from intracellular soluble protein extracts as well as protein-loaded beads were separated by 1D SDS PAGE (12% SDS gels) at 120 V. Proteins were in-gel digested using trypsin [59]. Peptides were separated by reversed phase chromatography and analyzed in an LTQ-Orbitrap Classic mass spectrometer equipped with a nanoelectrospray ion source [60]. MS/MS spectra were searched against a target decoy database using MaxQuant v. 1.6.10.43 [61]. The database covered all protein sequences predicted from the *B. licheniformis* DSM13 genome, selected *F. agariphila* KM3901^T^ ulvan PUL-encoded enzymes (Additional file 1: Table S1) and common laboratory contaminants as well as corresponding reversed sequences (decoys). The MaxQuant computed iBAQ values (intensity-based absolute quantification [62]) were used to manually calculate %riBAQ values, giving the relative protein abundance in % per sample. Quantified *F. agariphila* KM3901^T^ proteins were excluded from %riBAQ calculations. Proteins quantified in at least two out of the three replicates were considered for further calculations and for statistical tests. Since the total number of quantified proteins varied considerably between substrates (e.g., 933 proteins in ulvan extracellular samples compared to 247 proteins in the UHB samples), %riBAQ mean values were ranked according to their abundance (e.g., rank 1 for most abundant protein in the sample) to increase comparability between conditions. In addition, the total number of quantified proteins per sample was considered for the color code in graphs. Welch’s two-sided t-test (permutation-based FDR 0.05) identified statistical significance to protein abundance differences between samples within the intracellular soluble proteome samples and within the extracellular samples using Perseus v. 1.6.0.7 [63]. Only samples with a similar number of quantified proteins were compared. CAZymes were identified using dbCAN2 [37]. The enrichment of protein fractions is not exclusive and overlaps may occur, e.g., due to cell lysis or intracellular production of extracellular enzymes. Therefore, protein localization was also predicted using PSORTb v3.0.2 [38]. Proteomic data were deposited to the ProteomeXchange Consortium via the PRIDE partner repository [64] with the dataset identifier PXD033411. CAZymes were identified using dbCAN2 [37].

### Development of a *Bacillus* host-vector system

The nucleotide sequence of both genes from *F. agariphila* KMM3901^T^ P30_PL28 and P33_GH105 were ordered by GenScript Biotech (Leiden, Netherlands). Both synthetic genes were codon-optimized for expression in *B. licheniformis* using the GenSmart™ Codon Optimization tool (GenScript). The algorithm utilizes a matrix for the most frequently occurring codons in *B. licheniformis*. The constructs were assembled from synthetic oligonucleotides and provided in the backbone of the pUC19 vector. Amplification of the PL28 nucleotide sequence with *csn* and 00338 signal peptides (SP) was carried out in two discrete polymerase chain reactions, first with oligonucleotides MaZu8 and MaZu9 and second with MaZu7 and MaZu9 for the *csn*-SP whereas fusion of the 00338-SP was carried out with MaZu13/MaZu9 and MaZu12/Mazu9. The nucleotide sequence of GH105 with *csn*-SP was amplified with oligonucleotides MaZu10 and MaZu11 in a first PCR and, using the purified PCR product as the template, with MaZu7 and Mazu11 in a second PCR. Fusion of the 00338-SP to the GH105 sequence occurred with oligonucleotides MaZu14/MaZu11 and MaZu12/MaZu11. The PCR products were digested with *Nde*I and *Kpn*I and subsequently gel-purified. After ligation into the *Nde*I and *Kpn*I sites of pMSE3 *P*_*apr*_, *E.* *coli* DH10B was transformed with the recombinant plasmids, yielding pMSE3 P_*apr*_* csn*-UL, pMSE3 P_*apr*_ 00338-UL, pMSE3 P_*apr*_* csn*-GH and pMSE3 P_*apr*_ 00338-GH. Sequence identity of all expression vectors was verified by sequencing (Eurofins Genomics, Ebersberg, Germany). All four expression vectors were then transferred into both *Bacillus* expression hosts, *B. subtilis* JK138 and *B. licheniformis* MW3 [50] by electroporation.

Protein expression experiments were performed under simulated fed-batch conditions in EnpressoB-Medium as recommended by the manufacturer (Biosilta) at 30 °C and 250 rpm. Samples for protein analysis (SDS-PAGE, Activity screening and carbohydrate electrophoresis [15] were taken after 24 h and 48 h of cultivation.

### Development of a co-expression host *B. licheniformis* SH006

Construction of the appropriate expression vector was achieved by Gibson assembly. To this purpose, PCRs of the PL28 and GH105 expression cassettes were carried out with oligonucleotides MaZu37/MaZu29 (PL28) and MaZu28/MaZu35 (GH105) using pMSE3 P_*aprE*_* csn*-UL and pMSE3 P_*aprE*_ 00338-GH as the templates. The vector backbone of the medium copy-vector pBE-S was amplified with oligonucleotides MaZu38/MaZu36. The PCR products were gel-purified and, in case of the pBE-S vector backbone, digested with *Dpn*I in order to remove remaining circular plasmid DNA. All purified DNA fragments were then assembled in a vector:insert ratio of 1:2 and 3 µL of the reaction were used for transformation of *E. coli* DH10B yielding pBE-S P_*aprE*_* csn*-UL - P_*aprE*_ 00338-GH (pBE-S PL28-GH105). Sequence identity of the PL28-GH105 co-expression vector was verified and the plasmid was subsequently integrated into *B. licheniformis* SH006 by electroporation. Protein expression experiments of the newly constructed *B. licheniformis* SH006 PL28-GH105 co-expression strain were performed as before for the single constructs. Additionally, in order to demonstrate the ability of the newly constructed *B. licheniformis* SH006 PL28-GH105 strain to grow on ulvan, cultivations in M9-mineral media supplemented with either d-glucose or ulvan as the sole carbon source and also without any carbon source were carried out as described before.

### Activity measurement of ulvan lyase (PL28) and glycoside hydrolase (GH105)

The ulvan lyase activity was detected as described before [15] using the intra- or extracellular extract of *Bacillus* sp. cultivations instead of purified protein. For the detection of the glycoside hydrolase (GH105) activity the reversed ulvan lyase assay was used, while ulvan was PL28-pre-hydrolysed and heat inactivated after 16 h. The breakdown products resulting from the ulvan lyase assays were additionally analyzed via C-PAGE, the MBTH- assay and the thiobarbituric acid assay as described before [15]*.*

### Construction of the plasmid libraries for PL28 and GH105 activity screening

In order to obtain both plasmid libraries with 173 different types of signal peptides DNA sequences in the required size of at least 2000 *E. coli* clones, the “*B. subtilis* Secretory Protein Expression System” (Takara/Clontech) in combination with the “In Fusion HD Cloning Plus Kit” (Takara/Clontech) was used according to the manufacturer’s instructions. To this end, the nucleotide sequences for PL28 and GH105 were amplified from pMSE3-PL28 and pMSE3-GH105 using oligonucleotides MaZu19/MaZu20 (for PL28) and MaZu21/MaZu22 (for GH105). After restriction with *Nde*I and *Xba*I, the purified PCR products and the pBE-S vector were ligated and *E. coli* DH10B was transformed with the recombinant plasmids pBE-S-PL28 and pBE-S-GH105. After validation of sequence identity for PL28 and GH105, all different signal peptide sequences included in the provided SP library were integrated into the vector backbones of pBE-S-PL28 and pBE-S-GH105 following the manufacturer’s instructions. In brief, the *Eag*I and *Mlu*I digested vector was ligated with the 173 SP-containing DNA mixture using the “In Fusion Cloning” technology. Chemically competent *E. coli* Stellar cells (included in the kit) were transformed with 2 µL of the “In Fusion” reaction and selected on LB agar plates with ampicillin. All colony forming units (cfu) were rinsed from the plate to isolate the SP-plasmid library, which was subsequently integrated into the *Bacillus* expression hosts by electroporation.

## Supplementary Information

**Additional file 1**: **Table S1.** Proteins and accession numbers. **Table S2.** Bacterial strains. **Table S3.** M9-mineral media d-glucose composition. **Table S4.** M9-mineral media additives. **Table S5.** Primer list. **Figure S1.** Sugar composition of the cultivation media. **Figure S2.** SDS-PAGE of *F. agariphila* KMM3901^T^ enzymes expressed recombinantly in *E. coli*. **Figure S3.** Consumption of 5-dehydro-4-deoxy-d-glucuronate from *B. licheniformis* DSM13 during cultivation. **Figure S4.** Growth of *B. licheniformis* DSM13 on different ulvan hydrolysates. **Figure S5.** *B. licheniformis* DSM13 CAZyme repertoire and their expression. **Figure S6.** Alignment *B. licheniformis* DSM13 sulfatases with lipoteichoic acid synthases. **Figure S7.** Comparison of different *Bacillus* sp. to digest ulvan hydrolysate and ulvan derived monosaccharides. **Figure S8.** Growth curves of PL28 and GH105 *Bacillus* sp. expression strains. **Figure S9.** Activity assays from *Bacillus* sp. PL28 and GH105 expression strains. **Figure S10.** C-PAGE results from *Bacillus* sp. PL28 and GH105 expression strains. **Figure S11.** Growth of the different *B. licheniformis* expression strains. **Figure S12.** Activity assay results from *B. licheniformis* SH006, PL28 and GH105 single- and co-expression strain. **Figure S13.** C-PAGE results from *B. licheniformis* SH006 PL28, GH105 and co-expression strains. **Figure S14.** C-PAGE from the cultivation supernatant of *B. licheniformis* strains in M9-mineral media.

**Additional file 2: Table S6.** Summary of the proteomic results.

**Additional file 3: Table S7.** Results of statistical analyses.

## Data Availability

Proteomic data were deposited to the ProteomeXchange Consortium via the PRIDE partner repository [64] with the dataset identifier PXD033411.

## Abbreviations

CAZyme: Carbohydrate active enzyme
GH: Glycoside hydrolase
UL: Ulvan lyase
U: Ulvan
UH: Ulvan hydrolysate
UHA: Ulvan hydrolysate resulting from the digestion of ulvan with the enzymes P30_PL28 and P33_GH105
UHB: Ulvan hydrolysate resulting from the digestion of ulvan with the whole ulvan specific enzyme cascade according to Reisky et al. [14]
