# Supplementary material for: Metabolic engineering enables Bacillus licheniformis to grow on the marine polysaccharide ulvan
Source: Microb Cell Fact. 2022 Oct 10;21:207. doi: 10.1186/s12934-022-01931-0 (PMC9549685; doi:10.1186/s12934-022-01931-0)
Supplement: Supplementary file 1 — Additional file 1: Table S1. Proteins and accession numbers. Table S2. Bacterial strains. Table S3. M9-mineral media d-glucose composition. Table S4. M9-mineral media additives. Table S5. Primer list. Figure S1. Sugar composition of the cultivation media. Figure S2. SDS-PAGE of F. agariphila KMM3901T enzymes expressed recombinantly in E. coli. Figure S3. Consumption of 5-dehydro-4-deoxy-d-glucuronate from B. licheniformis DSM13 during cultivation. Figure S4. Growth of B. licheniformis DSM13 on different ulvan hydrolysates. Figure S5. B. licheniformis DSM13 CAZyme repertoire and their expression. Figure S6. Alignment B. licheniformis DSM13 sulfatases with lipoteichoic acid synthases. Figure S7. Comparison of different Bacillus sp. to digest ulvan hydrolysate and ulvan derived monosaccharides. Figure S8. Growth curves of PL28 and GH105 Bacillus sp. expression strains. Figure S9. Activity assays from Bacillus sp. PL28 and GH105 expression strains. Figure S10. C-PAGE results from Bacillus sp. PL28 and GH105 expression strains. Figure S11. Growth of the different B. licheniformis expression strains. Figure S12. Activity assay results from B. licheniformis SH006, PL28 and GH105 single- and co-expression strain. Figure S13. C-PAGE results from B. licheniformis SH006 PL28, GH105 and co-expression strains. Figure S14. C-PAGE from the cultivation supernatant of B. licheniformis strains in M9-mineral media. [file 12934_2022_1931_MOESM1_ESM.pdf]

Additional file 1_ supplementary table and figures

Metabolic engineering enables *Bacillus licheniformis* to grow on the marine polysaccharide ulvan

Theresa Dutschei[1], Marie-Katherin Zühlke[2,3], Norma Welsch[2,3], Tom Eisenack[2,] Maximilian Hilkmann[2,3], Joris Krull[3,4,5], Carlo Stühle[1], Stefan Brott[1], Alexandra Dürwald[2], Lukas Reisky[1], Jan-Hendrik Hehemann[3,4,5], Dörte Becher[6], Thomas Schweder[2,3‡] and Uwe T. Bornscheuer [1,3‡]

[1]Department of Biotechnology & Enzyme Catalysis, Institute of Biochemistry, University of Greifswald, 17487 Greifswald, Germany

[2]Department of Pharmaceutical Biotechnology, Institute of Pharmacy, University of Greifswald, 17487 Greifswald, Germany

[3]Institute of Marine Biotechnology e.V., 17489 Greifswald, Germany

[4]Max Planck-Institute for Marine Microbiology, 28359 Bremen, Germany

[5]University of Bremen, Center for Marine Environmental Sciences (MARUM), 28359 Bremen, Germany

[6]Department of Microbial Proteomics, Institute for Microbiology, University Greifswald, 17487 Greifswald, Germany

‡ Corresponding authors:

Thomas Schweder, schweder@uni-greifswald.de

Uwe T. Bornscheuer, uwe.bornscheuer@uni-greifswald.de

Additional tables

- Table S1: Proteins and accession numbers
- Table S2: Bacterial strains and plasmids
- Table S3: M9-mineral media D-glucose composition
- Table S4: M9-mineral media additives
- Table S5: Primer list
- The Supplementary Tables S6 and S7 are available as separate Excel files.

Additional figures

- Figure S1: Sugar composition of the cultivation media
- Figure S2: SDS-PAGE of *F. agariphila* KMM3901^T enzymes expressed recombinantly in *E. coli*
- Figure S3: Consumption of 5-dehydro-4-deoxy-D-glucuronate from *B. licheniformis* DSM13 during cultivation
- Figure S4: Growth of *B. licheniformis* DSM13 on different ulvan hydrolysates
- Figure S5: *B. licheniformis* DSM13 CAZyme repertoire and their expression
- Figure S6: Alignment *B. licheniformis* DSM13 sulfatases with lipoteichoic acid synthases
- Figure S7: Comparison of different *Bacillus* sp. to digest ulvan hydrolysate and ulvan derived monosaccharides
- Figure S8: Growth of the *Bacillus* sp. PL28 and GH105 expression strains
- Figure S9: Activity assays of *Bacillus* sp. PL28 and GH105 expression strains
- Figure S10: C-PAGE results from *Bacillus* sp. PL28 and GH105 expression strains.
- Figure S11: Growth of the different *B. licheniformis* expression strains.
- Figure S12: Activity assay results from *B. licheniformis* SH006, PL28 and GH105 single- and co-expression strain.
- Figure S13: C-PAGE results from *B. licheniformis* SH006 PL28, GH105 and co-expression strains.
- Figure S14: C-PAGE from the cultivation supernatant of *B. licheniformis* strains in M9-mineral media.

Table S1: List of *F. agariphila* proteins used to produce ulvan hydrolysates. (UHA) P30_PL28 and P33_GH105 and (UHB) all listed enzymes. All proteins were recombinantly expressed in *E. coli* as described previously [1].

Name	Locus tag	Uniprot ID	Functional annotation	size [kDa]
P1_GH88	*21900	T2KLZ3	Unsaturated glucuronylhydrolase (GH88)	44.1
P10_PLnc	*21990	T2KNA3	Ulvan lyase (PLnc)	92.6
P17_GH2	*22060	T2KN75	β-Galactosidase (GH2)	112.9
P18_S1_7	*22070	T2KPK5	Arylsulfatase (S1_7)	53.1
P20_GH78	*22090	T2KNB2	α-L-rhamnosidase (GH78)	100.4
P24_GH3	*22130	T2KMH0	β-Glucosidase (GH3)	79.3
P27_GH43	*22160	T2KN85	β-Xylosidase (GH43)	67.3
P30_PL28	*22190	T2KNC2	Ulvan lyase (PL28)	44.1
P31_GH39	*22200	T2KM23	Glycoside hydrolase (GH39)	54.6
P33_GH105	*22220	T2KPL9	Glycoside hydrolase (GH105)	40.7
P36_S1_25	*22250	T2KM26	α-L-rhamnosidase/-sulfatase (GH78/S1_25)	134.3
	*=BN863			

Table S2: Bacterial strains and plasmids

**Strain**	**Description or genotype**	**Reference or source**
Escherichia coli TOP10	*F- mcrA* Δ(mrr-hsdRMS-mrcB) φ80lacZΔM15 Δ*lacX74 nupG* recA1 araD139 Δ(ara-leu)7697 galK16 rpsL(StR) endA1 fhuA2 λ-	Originally purchased from Invitrogen by Thermo Fisher Scientific (Waltham, MS, USA)
Escherichia coli BL21(DE3)	B F-- ompT gal dcm lon hsdSB(rB—mB-) λ(DE3 [lacI lacUV -T7 gene 1 ind1 sam7 nin5])	Originally purchased from New England Biolabs (Ipswich, MS, USA)
Bacillus subtilis B1	Wildtype	Department of Biotechnology & Enzyme Catalysis (University of Greifswald)
Bacillus licheniformis DSM13	Wildtype	Department of Pharmaceutical Biotechnology (University of Greifswald) originating from Veith et al [2]
Staphylococcus carnosus 80-285	Wildtype	Kindly provided by the Enzymicals AG (Greifswald, Germany)
Staphylococcus carnosus 20-282	Wildtype	Kindly provided by the Enzymicals AG (Greifswald, Germany)
Saccharomyces cerevisiae GRF18	Wildtype	Department of Biotechnology & Enzyme Catalysis (University of Greifswald)
Vibrio natriegens ATCC 14048	Wildtype	Originating from the ATCC (Manassas, V, USA)
Pseudomonas putida DSMZ 50198	Wildtype	Originating from the DSMZ (Braunschweig, German)
Pichia pastoris X33	Wildtype	Originally from Invitrogen by Thermo Fisher Scientific (Waltham, MS, USA)
Cutaneotrichosporan curvatus DSM 101032	Wildtype	Department of Computational Synthetic Biology (TU Darmstadt) as described by Hofmeyer *et al.* [3]
Cupriavidus necator H16	Wildtype	Department of Microbial proteomics (University of Greifswald) as described by Pohlmann *et al.* [4]
Bacillus licheniformis MW3	ΔhsdR1, ΔhsdR2	Waschkau *et al.* 2008 [5]
Bacillus subtilis JK138	*sfp*+, Δ*sacA::SpecR*, Δ*lytC::lox72*, Δ*bpr-spo::lox72*, Δ*nprB::lox72*, Δ*mpr::lox72*, Δ*aprE::lox72*, Δ*nprE::lox72*, Δ*vpr::lox72*, Δ*epr::lox72*, Δ*wprA::lox72,* Δ*srfA::(comS,lox72*), Δ*pksX::lox72*, Δ*pps::lox72*, Δ*amyE::lox72*	Krüger *et al.* 2022
Bacillus licheniformis SH006	Δ*hsdR1*, Δ*hsdR2*, Δ*pga*, Δ*apr*	this study
pE194SV	pE194 derivative; *E. coli* / *Bacillus*-shuttle vector; Em*R*; ori pE194; AmpR, ori pUC18	This work
pDhsdR1	pE194SV with Δ*hsdR1* homology flanking region	This work
pDhsdR2	pE194SV with Δ*hsdR2* homology flanking region	This work
pDpga	pE194SV with Δ*pga* homology flanking region	This work
pDapr	pE194SV with Δ*apr* homology flanking region	This work

Table S3: D-glucose-supplemented M9-mineral medium

**M9 - mineral media stock solution**	**Final concentration per 1 L**
M9 salt solution	1x
20 % glucose	0.4 %
1 M $MgSO_4$	1 mM
1 M $CaCl_2$	0.3 mM
Biotin (1 mg/ml)	4.09 nM
Thiamine (1mg/ml)	3.77 nM
Trace element solution (100x)	1 x

Table S4: M9-mineral medium additives

Additive	Component	Concentration in stock solution
M9 salts (10x)	Na_2HPO_4 *2 H_2O<br>KH_2PO_4<br>NaCl<br>NH_4Cl<br>ddH_2O	422.6 mM<br>141.4 mM<br>93.6 mM<br>93.5 mM<br>1 L
Ulvan solution	Ulvan<br>Sodium phosphate buffer<br>Sodium chloride<br>pH 7.5	2 %<br>25 mM<br>50 mM
Ulvan hydrolysate	Ulvan<br>Sodium phosphate buffer<br>Sodium chloride<br>pH 7.5<br>Enzyme mix	2 %<br>25 mM<br>50 mM<br><br><br>5 µL/mL per enzyme
Enzyme mix	Sodium phosphate buffer<br>Sodium chloride<br>pH 7.5<br>Enzyme mix	25 mM<br>50 mM<br><br>5 µL/mL per crude extract enzyme from table S1
Trace element solution 100 x	$FeCl_3$<br>$ZnCl_2$<br>0.1 M $CaCl_2$ • 2$H_2$0<br>0.2 M $CoCl_2$ • 6 H_2O<br>0.1 M HBO_3<br>1 M $MnCl_2$ • 4 $H_2$0<br>KI<br>Na_2MoO_4<br>EDTA	13.1 mM<br>0.62 mM<br>76 µM<br>42 µM<br>162 µM<br>8.1 µM<br>0.5 mM<br>1 mM<br>13.4 mM
Yeast extract		2 % (w/v)
Sodium chloride		15 % (w/v)

Table S5: List and sequences of primers used in this study.

Primer	Sequence 5´→ 3´
MaZu7	ACGTCATATGAAAATCAGTATGCAAAAAGCAGATTTTTGGAAAAAAGCAGCGATCTCATTACTTGTTTTCACCATGTTTTTTACCCTGATGATGAGCG
MaZu8	CACCATGTTTTTTACCCTGATGATGAGCGAAACGGTTTTTGCGCAAACGGCACCGGATGAAGACACAAGCGCCATTACGAG
MaZu9	ACGTGGTACCTTACAGGCTTTCGACTGCGATGGCTTTCCAGACGC
MaZu10	CACCATGTTTTTTACCCTGATGATGAGCGAAACGGTTTTTGCGCAAAAAGGCCTTAACCATAGCGAAATCGAAGC
MaZu11	ACGTGGTACCTTATTCTTCCAGTTTCAAGACTTCGCTTCCTGCCATCAG
MaZu12	ACGTCATATGTTGATCAACAAAAGCAAAAAGTTTTTCGTTTTTTCTTTCATTTTTGTTATGATGCTGAGCCTCTCATTTGTGAATGGGG
MaZu13	GATGCTGAGCCTCTCATTTGTGAATGGGGAAGTTGCAAAAGCCCAAACGGCACCGGATGAAGACACAAGCGCC
MaZu14	GATGCTGAGCCTCTCATTTGTGAATGGGGAAGTTGCAAAAGCCCAAAAAGGCCTTAACCATAGCGAAATCGAAGCG
MaZu19	ACGTACCATATGCAAACGGCACCGGATGAAGACACAAGCGCC
MaZu20	ACGTACTCTAGACAGGCTTTCGACTGCGATGGCTTTCCAGAC
MaZu21	ACGTACCATATGCAAAAAGGCCTTAACCATAGCGAAATCGAAGCG
MaZu22	ACGTACTCTAGATTCTTCCAGTTTCAAGACTTCGCTTCCTGCCATCAG
MaZu28	TGATAAGCGTTGGTTTGGCAATCTTATCGGGCTATGCATTTATAAAATG
MaZu29	GCATAGCCCGATAAGATTGCCAAACCAACGCTTATCAATAGAAAAAGAGCATTTTTTGAAACAAAACTTC
MaZu35	TGCGTTAGCAATTTAACTGTGATAAACTACCGCATTAATAGAAAAAGAGCATTTTTTGAAACAAAACTTC
MaZu36	TAATGCGGTAGTTTATCACAGTTAAATTGCTAACGCAGTCAGGCACCGT
MaZu37	GTTTTTAAAGGCTTTTAAGCCGTCTGTACGTTCCTAACATCTGATGTCTTTGCTTGGCGAATGTTCATCT
MaZu38	TTAGGAACGTACAGACGGCTTAAAAGCCTTTAAAAACGTTTTTAAGGGGTTTGTAGACAAGGTAAAGGATAAAACAG
P1-hsdR1	CTGCAGGGTCTCAACCCGAACAGCGTAAGGCTGATG
P2-hsdR1	CCGTAATTTGAATCTATTAGACAAACATCTTTTGTAGGAATG
P3-hsdR1	GATGTTTGTCTAATAGATTCAAATTACGGGCCTTG
P4-hsdR1	TCTAGAGGTCTCATGAGATCGGTTTTATGAAAGCGTC
P1-hsdR2	CTGCAGCGTCTCAACCCGATAAAAGGATTACTGTGCG
P2-hsdR2	TCCATGTTGTCACAACCTATTGTTGAGAATAAAGGAAAAGGAG
P3-hsdR2	CCTTTATTCTCAACAATAGGTTGTGACAACATGGAAAG
P4-hsdR2	TCTAGACGTCTCATGAGGTGCTTTCATCAATCGTAAATC

Table S6: Summary of the proteomic results (available as separate Excel file)

Table S7: Results of statistical analyses (Welch's T-test, FDR 0.05) (available as separate Excel file)

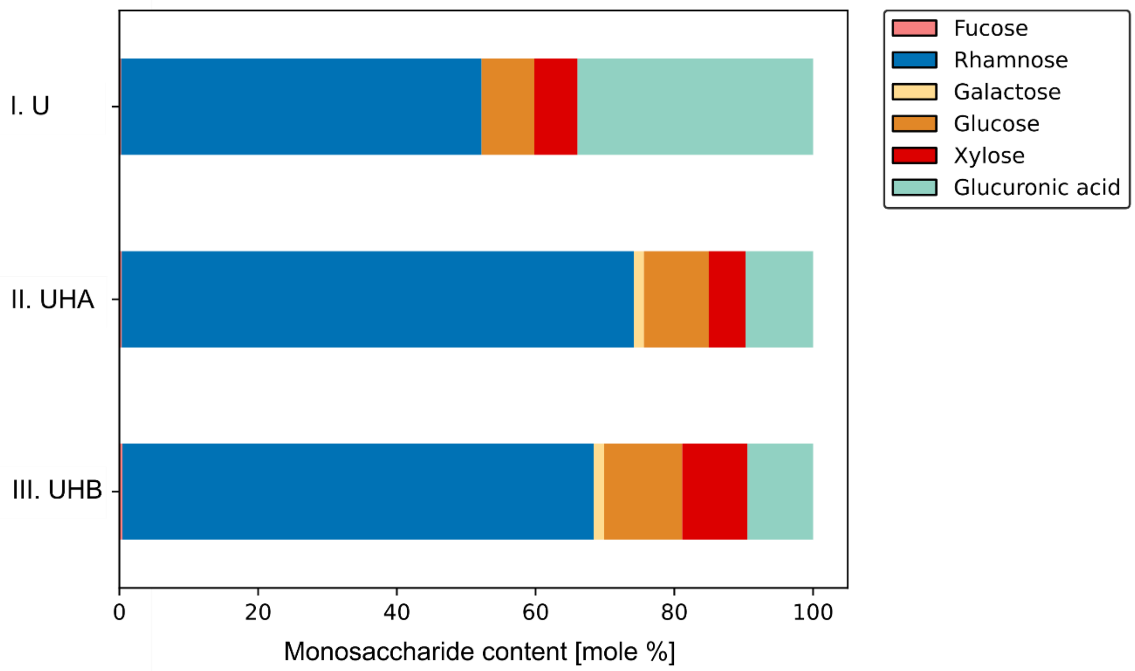

Fig. S1 Sugar composition of the cultivation media. The complex carbon sources were analysed for their detailed monosaccharide composition via acid hydrolysis and HPAEC-PAD. Ulvan (U), the partially P30_PL28 and P33_GH105-hydrolyzed ulvan (UHA) and the completely digested ulvan (UHB) using the whole cascade of ulvan degrading enzymes [1].

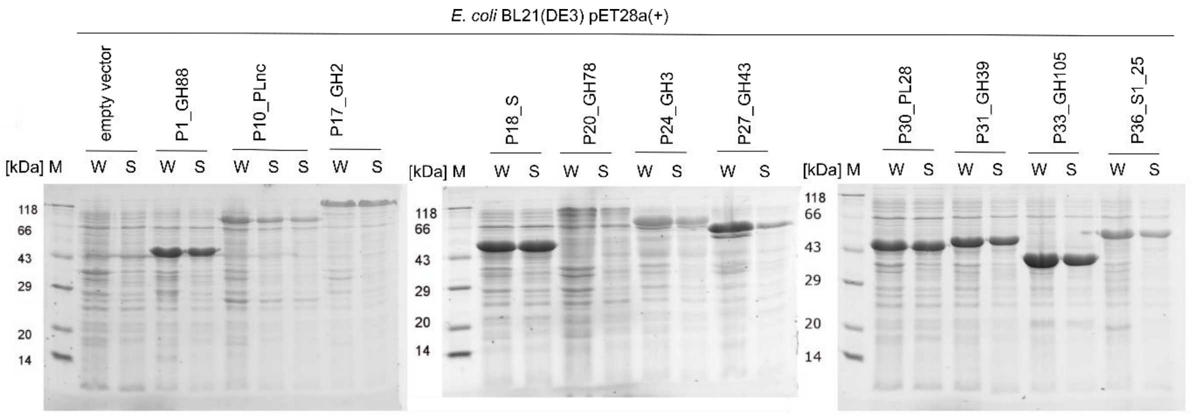

Fig. S2 SDS-PAGE of *F. agariphila* KMM3901^{T} enzymes expressed recombinantly in *E. coli* BL21(DE3) as described by Reisky *et al.* [1]. The cells were normed (7/OD) and the whole cell (W) extract and soluble protein (S) fraction were analyzed by SDS-PAGE containing 1% (V/V) trichloroethanol (TCE). The protein bands were visualised under UV and the pictures were colour-inverted and decolorized. The protein marker Roti®-Mark from Carl Roth (Karlsruhe, Germany) was used.

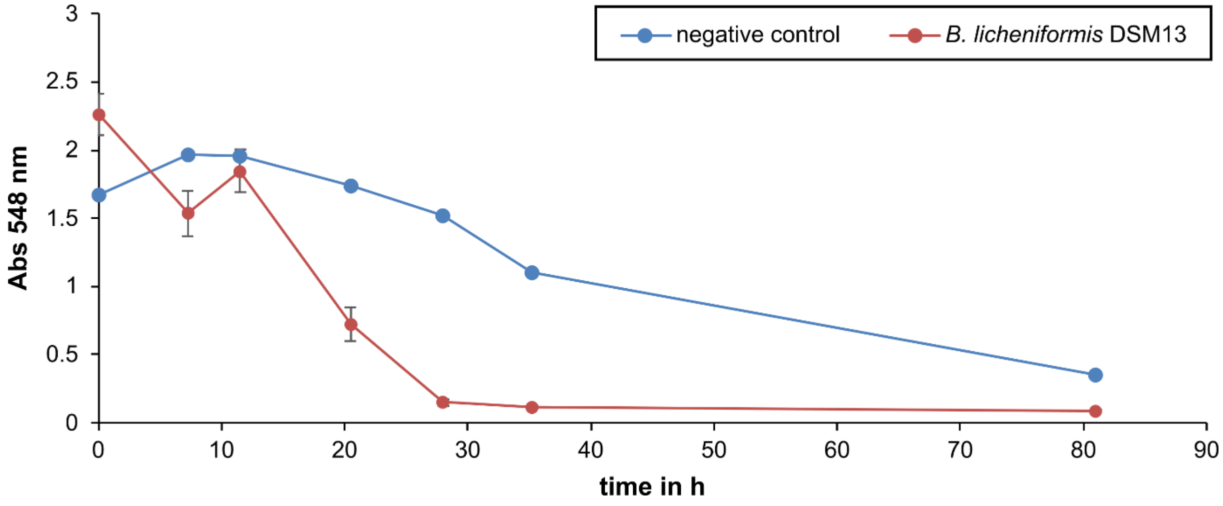

Fig. S3 Consumption of 5-dehydro-4-deoxy-D-glucuronate by *B. licheniformis* DSM13 during cultivation. Thiobarbituric acid assay determined 5-dehydro-4-deoxy-D-glucuronate in cultivation supernatants (M9-ulvan hydrolysate UHB, see Fig. 3a). Cell-free medium served as negative control.

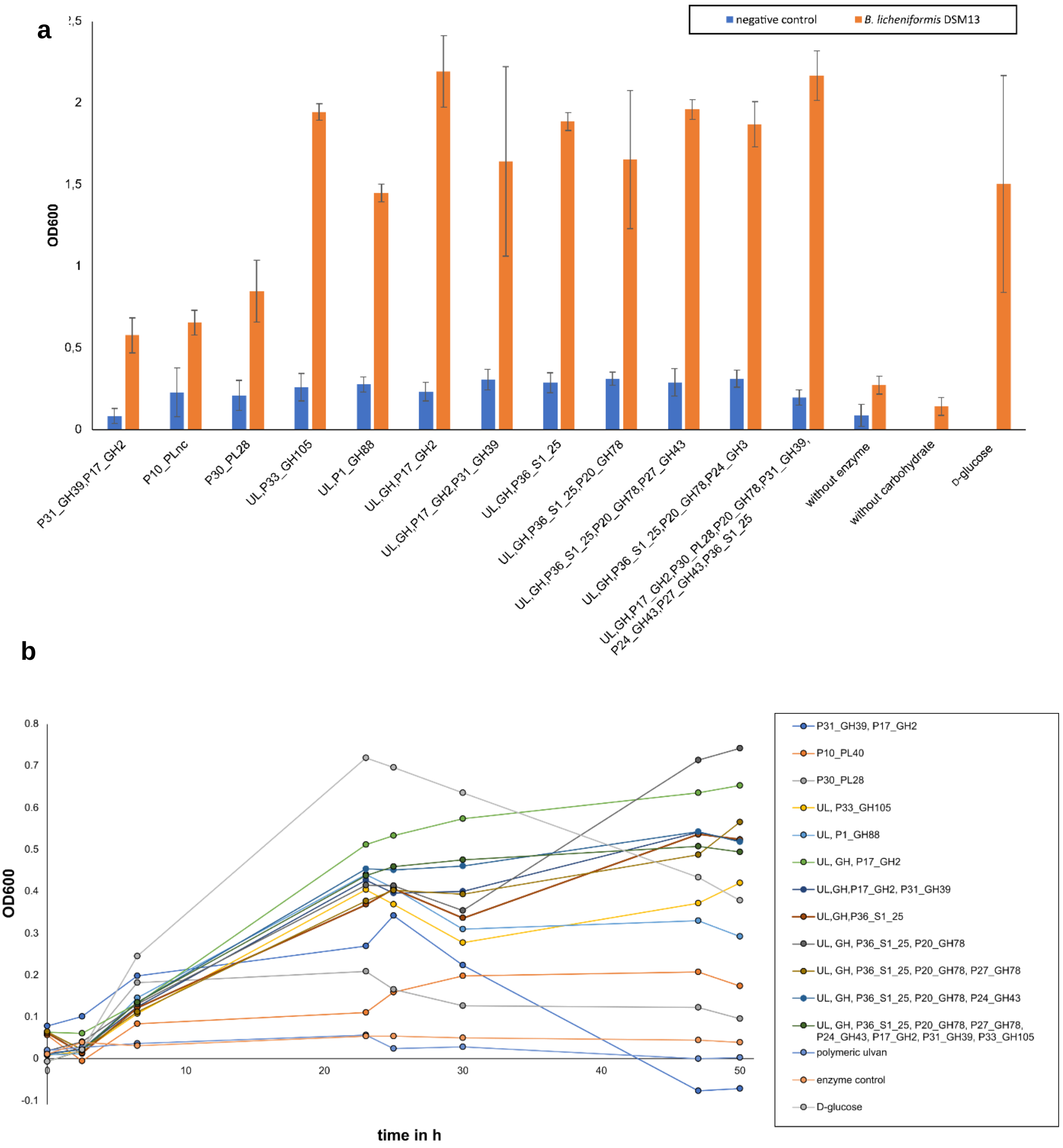

Fig. S4 Growth of *B. licheniformis* DSM13 on ulvan (without enzyme) and ulvan hydrolysates representing different levels of degradation. For hydrolysis, different *F. agariphila* enzymes, enzyme combinations or all enzymes (recombinantly expressed in *E. coli*) were used (Table S1). *B. licheniformis* DSM13 was cultivated in 1 mL in 96 deep-well plates and OD600 was measured after 48 h (**a**). This graph represents the full dataset of the Figure 2 in the main text. For investigation of the growth behaviour the culture was cultivated in 200 µL scale in a low-well plate and measured for 48 h (**b**).

	intracellular soluble					extracellular				
	Glucose	Rhamnose	Ulvan	UHA	UHB	Glucose	Rhamnose	Ulvan	UHA	UHB
PL9_2(33-390)/PL9/PL9_2/ **Q65IY0**	1253			1154	1196	639	234			
PL3_1(30-200)/PL3+CBM84/PL3_1/ **Q65EF5**			1351			223	14	35	12	198
PL26(6-841)/N/PL26/ **Q62WC4**										
PL11(40-621)/CE12+PL1 1/PL11_1/ **Q65KY4**								933		
PL11(23-624)/CE12+PL1 1/PL11_1/ **Q65KY1**										
PL1_8(223-417)/PL1/PL1_8/ **Q65G96**						895				
PL1_6(142-351)/PL1/PL1_6/ **Q65KV6**										
PL1_5(98-274)/PL1/PL1_5/ **Q65DC2**			1216	938		35	4	61	72	
N/GH23/GH23/ **Q65L19**										
N/GH23/GH23/ **Q65ET5**										
N/GH23/GH23/ **Q62U32**										
N/GH0/GH0/ **Q65IU8**	711	360	371	300	490		807		591	
GH9(42-475)+CBM3(502-586)/GH9+CBM3/CBM3+GH9/ **Q65JI9**										
GH73(731-858)/GH73/GH73/ **Q65E77**	428	1473	1346	1158	1189	86	471	117	81	6
GH73(11-150)/GH73/GH73/ **Q65ET7**										
GH68(39-478)/GH68/GH68/ **Q65EI8**	1434					431	639	748	146	72
GH65(323-693)/GH65/GH65/ **Q65MW3**		1365	339	293				182	52	
GH53(50-413)/GH53/GH53/ **Q65CX5**						429	72	169	199	
GH51(3-501)/GH51/GH51/ **Q65GC7**		990	822	636				799	592	
GH5_7(66-340)/GH5/GH5_7/ **Q65JI6**										
GH5_4(71-331)+CBM46(368-447)/GH5/GH5_4/ **Q65JI7**										
GH5_2(51-289)+CBM3(372-452)/GH5+CBM3/CBM3+GH5_2/ **A5A671**						208	196	758		
GH48(4-461)/GH48/GH48/ **Q65JI8**	1475	1507	705	644	1115		524	769		190
GH43_5(48-326)/GH43/GH43_5/ **Q65L63**						370	538		278	132
GH43_5(40-318)/GH43/GH43_5/ **Q65GB9**			836	197			469	103	28	
GH43_4(40-360)/GH43/GH43_4/ **Q65D31**		1482	629	479		232	15	22	8	
GH43_12(8-289)/GH43/GH43_12/ **Q65MB6**										
GH43_10(27-294)/GH43/GH43_10/ **Q65MB7**										
GH42(6-385)/GH42/GH42/ **Q65CX4**	1419	1426						757	530	
GH42(20-394)/GH42/GH42/ **Q65NG1**	1388	647	873	820		724	320	595		
GH42(10-369)/GH42/GH42/ **Q65KX8**		1414				474	622	798		
GH4(7-184)/GH4/GH4/ **Q65MC5**		588	301	374			817	404	328	
GH4(6-185)/GH4/GH4/ **Q65DG5**	1336	585	594	641				885		
GH4(4-183)/GH4/GH4/ **Q65NS1**										
GH4(3-181)/GH4/GH4/ **Q65LL3**		1512	1128			846	508	596	351	
GH32(46-346)/GH32/GH32/ **Q65EI7**										
GH32(39-349)+CBM66(520-673)/GH32+CBM66/CBM66+GH32/ **Q65GW7**						277	129	118	56	
GH32(32-337)/GH32/GH32/ **Q65DN5**			976			767		373	612	
GH32(27-330)/GH32/GH32/ **Q65D73**						822	707			
GH31(244-690)/GH31/GH31/ **Q65IV9**										
GH31(236-665)/GH31/GH31/ **Q65EZ5**										
GH3(74-305)+CBM6(863-981)/GH3+CBM6/CBM6+GH3/ **Q65EZ4**	1412	1552		924			840			
GH3(112-349)/GH3/GH3/ **Q65P58**	1477	1130	615	324		675	327	172	139	
GH28(50-407)/GH28/GH28/ **Q65F26**						448	175	540	401	
GH26(30-348)/GH26/GH26/ **Q65MP4**										
GH25(6-175)+CBM50(218-261)/GH25/CBM50+GH25/ **Q65KM7**				1096		789	672	882		
GH23(112-215)/GH23/GH23/ **Q65LA6**										
GH18(68-521)/GH18/GH18/ **Q65NR7**						862	857	765	611	
GH18(40-437)+CBM5(549-588)/GH18/CBM5+GH18/ **A5A699**			1219	1116		723		310	356	
GH18(178-400)/GH18+CBM50/CBM50+GH18/ **Q62US6**	1469		987	849		288	344	342	96	
GH18(167-416)/GH18+CBM50/CBM50+GH18/ **Q65PK4**	842	354	121	181		164	154	176	48	
GH13_5(61-398)/GH13/GH13_5/ **Q65MX0**						779	405	180	97	
GH13_31(28-371)/GH13/GH13_31/ **Q65FM9**						472	599	745		
GH13_31(27-377)/GH13/GH13_31/ **Q65MW2**		1099	191	138				480	243	
GH13_29(30-375)/GH13/GH13_29/ **Q65MI2**		581	866	678	853				396	167
GH126(45-353)/GH126/GH126/ **Q65D19**										
GH12(108-260)/GH12/GH12/ **Q65FM6**						130	140	516	327	195
GH105(30-363)/GH105/GH105/ **Q65FY8**										
GH105(25-336)/GH105/GH105/ **Q65KY9**										
GH1(8-475)/GH1/GH1/ **Q65NL9**	1413	1474	1396			183	179	572	533	
GH1(7-468)/GH1/GH1/ **Q65D52**	872	546	835	929	1156	316	303	238	331	
GH1(7-468)/GH1/GH1/ **Q65D83**										
GH1(5-484)/GH1/GH1/ **A5A692**	1228	1381				479	568	601		
GH1(5-466)/GH1/GH1/ **Q65D37**	663	159	234	298	940	614	223	347	429	148
GH1(12-470)/GH1/GH1/ **Q65HN0**			1008	1039		689	631	308	218	
CE9(12-390)/CE9/CE9/ **Q65CQ8**										
CE8(11-310)/CE8/CE8/ **Q65F39**		258	350	387						
CE7(5-314)/CE7/CE7/ **Q65NM7**	648	584	735	565	691	638		518	336	
CE6(55-146)/CE6/CE6/ **Q65GZ2**	458	454	1195	1068	659	343	475	630	435	
CE4(75-179)/CE4/CE4/ **Q65HY6**										
CE4(59-185)/CE4/CE4/ **Q62XU4**			1001							
CE4(51-179)/CE4/CE4/ **Q65P65**	1039	502	413	256			733	775		
CE4(267-380)/CE4/CE4/ **Q62YB4**							830	854		42
CE4(125-247)/CE4/CE4/ **Q62UX9**	1288	900	999	979		389	554	375	163	
CE4(115-241)/CE4/CE4/ **Q62NS5**										
CE4(100-227)/CE4/CE4/ **Q65LW7**								767		39
CE14(6-124)/CE14/N/ **Q65IG8**	1309	1257			1154					
CE14(17-122)/CE14/CE14/ **Q62TK1**	923	897	1156		998	518	293	667		
CE12(7-215)/CE12/CE12/ **Q62WC9**										
CE12(6-211)/CE12/CE12/ **Q62NX1**	1321	1417								
CE12(5-202)/CE12/CE12/ **Q65KX9**	437	466	508	625	586		666			
CE12(11-216)/CE12/CE12/ **Q65KY7**										
CBM68(6-98)+GH13_14(261-565)/GH13+CBM68/CBM48+CBM68+GH13_14/ **Q65G09**							855			
CBM63(139-214)/GH5+CBM63/CBM63/ **Q65IG3**						112	122	305	94	40
CBM48(26-109)+GH13_9(178-468)/GH13/CBM48+GH13_9/ **Q65FS4**										
CBM34(6-127)+GH13_20(176-469)/GH13+CBM34/CBM34+GH13_20/ **Q62YB1**			784	575				147	234	
highest rank	1	1	1	1	1	1	1	1	1	1
owest rank	1498	1559	1414	1197	1215	904	857	933	629	247

abundance rank: high – low

Fig. S5 List of PLs, GHs and CEs identified by dbCAN2 [6] and their abundance in the intracellular soluble and extracellular proteomes. The graph indicates the relative abundance of proteins within the respective sample given as abundance ranks. Abundance ranks were derived from %riBAQ values (Table S6). The lowest rank corresponds to the total number of quantified proteins per sample. Blank tiles represent proteins that were not quantified. UHA/B: Ulvan hydrolysate A/B (see Fig. 3a). Protein IDs are highlighted in bold together with the full output of dbCAN2 analyses (HMMER/Hotpep/DIAMOND/Protein ID).

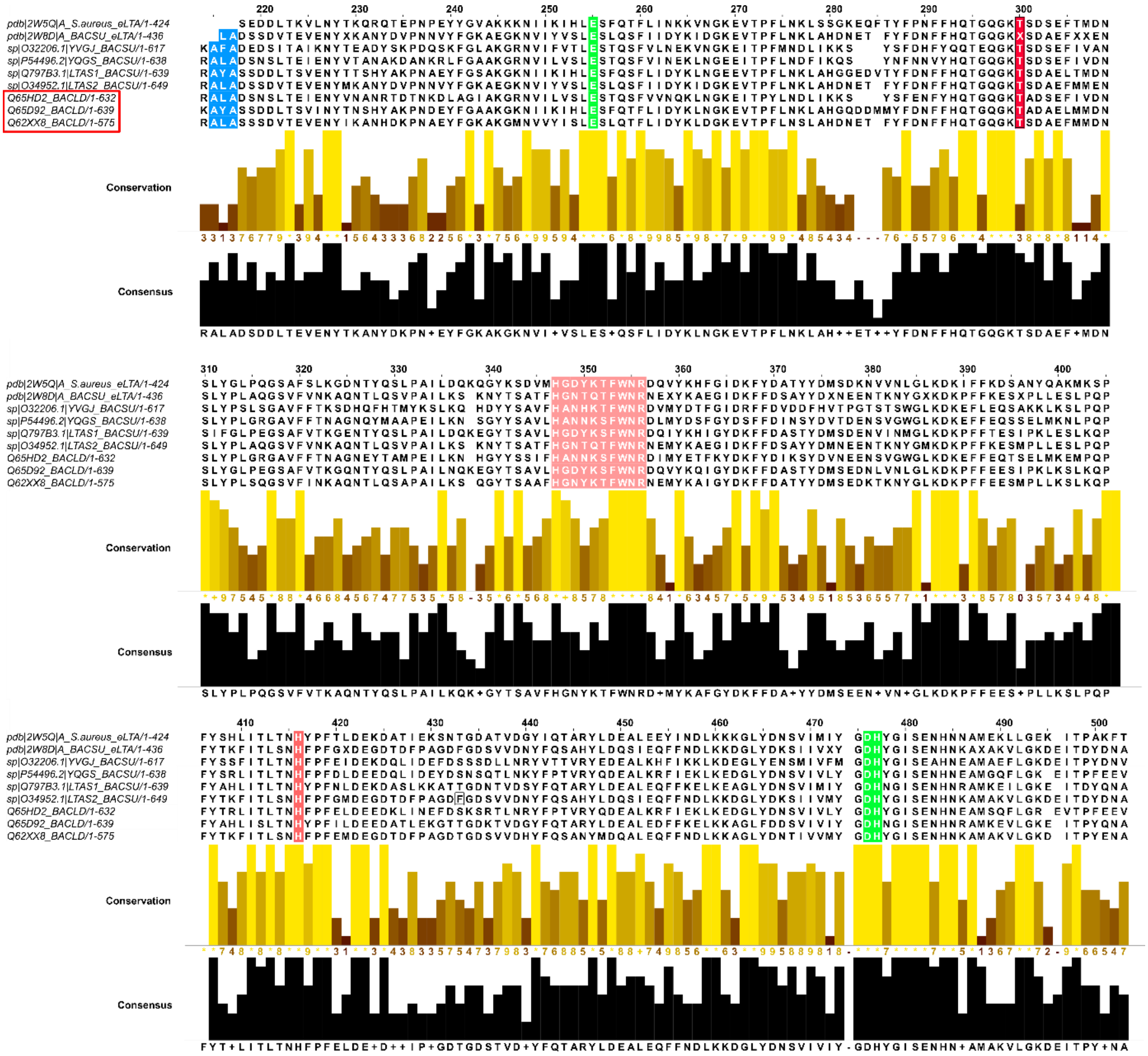

Fig. S6 Alignment of sulfatases from *B. licheniformis* DSM13 with lipoteichoic acid synthases of other Gram-positive bacteria. The lipoteichoic acid synthases (LTA) were the highest hit in the BlastP search. LTAs consist of a transmembrane and an extracellular domain. Amino acid residues in the region of the extracellular LTA (eLTA) domain (210 to 585 As) are displayed. LTAs are synthesized as membrane proteins and are cut at a cleavage sequence AXA (blue). T300 (marked in red) is the catalytic residue in the eLTA of *S. aureus*, while H416 is involved in the reaction mechanism (protonation of the leaving group). Residues marked in green are important for the binding of Mn2+. Residues 347-356 (HxD/NxxFW/YNR) are important for substrate binding [7].

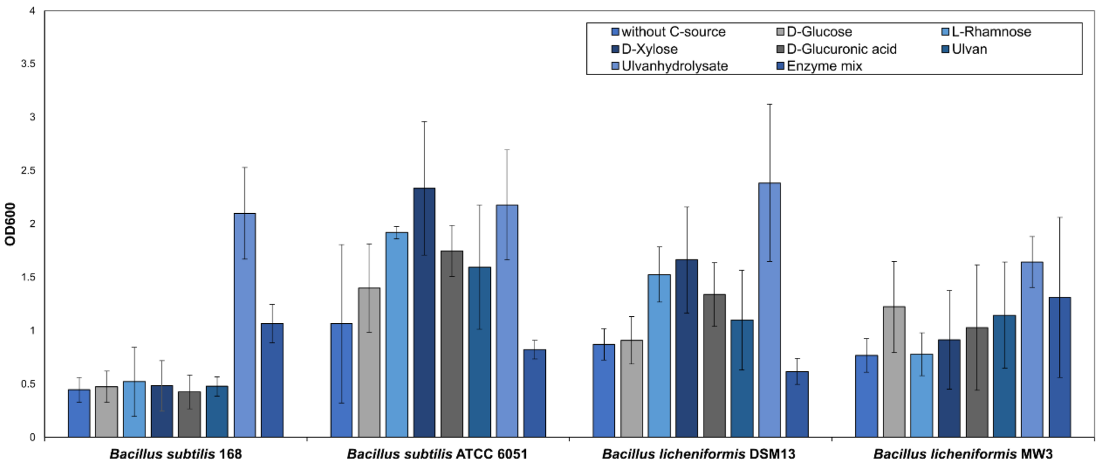

Fig. S7 Comparison of different *Bacillus* sp. for the conversion of ulvan hydrolysate and ulvan derived monosaccharides. The strains were cultivated in 1 mL Belitzky-Minimal media [8] with variation of the carbon source in a 96-deep well-plate. General growth in OD600nm was measured after 24 h, showing a general acceptance of the ulvan hydrolysate for all four *Bacillus* sp. in the chosen Belitzky-Minimal medium conditions.

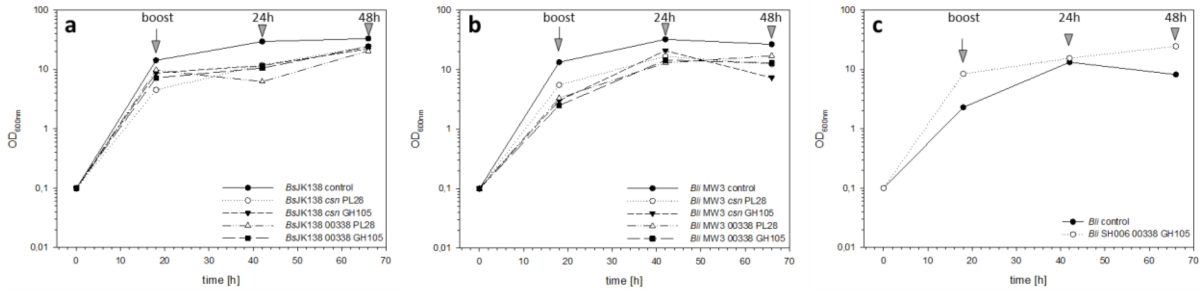

Fig. S8 Growth of the PL28 and GH105 *Bacillus* expression strains. *B. subtilis* JK138. (**a**), B. licheniformis MW3 (**b**) and *B. licheniformis* SH006 (Δapr) (**c**) were grown under simulated fed-batch conditions in Expresso-B medium at 30°C and 250 rpm. Sampling points for activity measurements, 24 h and 48 h after the boost are indicated.

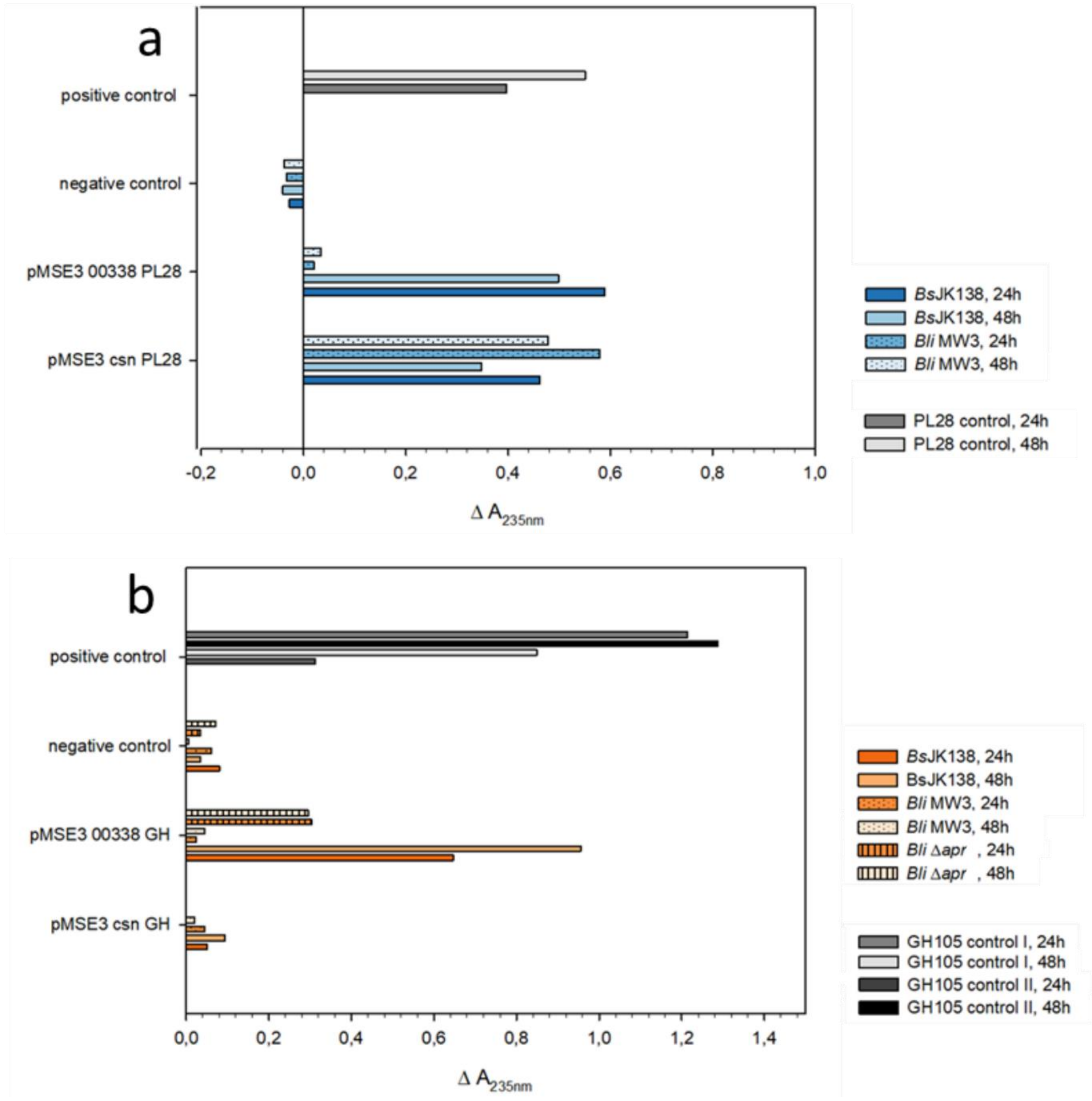

Fig. S9 Activity assay results from *Bacillus* sp. PL28 and GH105 expression strains. (**a**) Results of the ulvan lyase (UL) assay measurement of the lyase product formation at 235 nm over 60 min. The deviation of the absorption of the end-start reveals the lyase product formation (lyase activity), while the deviation of the absorption start-end shows the reversed reaction of the GH105 activity (**b**) cleaving of the lyase moiety using prehydrolyzed ulvan from the recombinantly expressed ulvan lyase PL28.

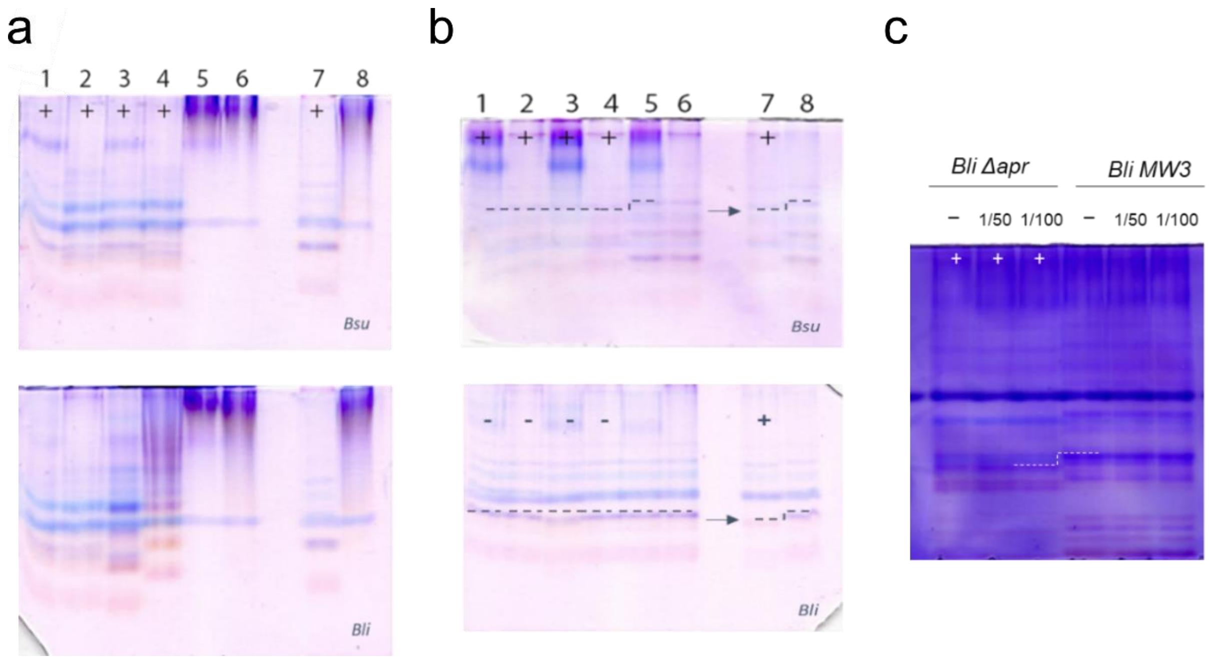

Fig. S10 C-PAGE results from *Bacillus* sp. PL28 and GH105 expression strains. (**a**) The C-PAGE (carbohydrate polyacrylamide gel electrophoresis) corresponds to the ulvan lyase assay shown in Fig. S10 to the results of the ulvan lyase (UL) expression and (**b**) reaction of the GH105 activity cleaving of the lyase moiety using prehydrolyzed ulvan from the recombinantly expressed ulvan lyase PL28. Intracellular (in) and extracellular (ex) fractions of *B. subtilis* JK138 (Bsu) and *B. licheniformis* DSM13 (Bli) were analyzed. Lane 1: TB1 csn-UL ex, Lane 2: TB1 csn-UL in, Lane 3: TB1 00338-UL ex, Lane 4: TB1 00338-UL in, Lane 5: Ko (empty vector) ex, Lane 6: Ko (empty vector), Lane 7: positive control, Lane 8: negative control. (**c**) C-PAGE after 24 h of GH105 expression in *B. licheniformis* SH006 (Bli Δapr) and *B. licheniformis* MW3 (Bli MW3) with the addition of the protease inhibitor in 1/100, 1/50 and no protease (−) addition.

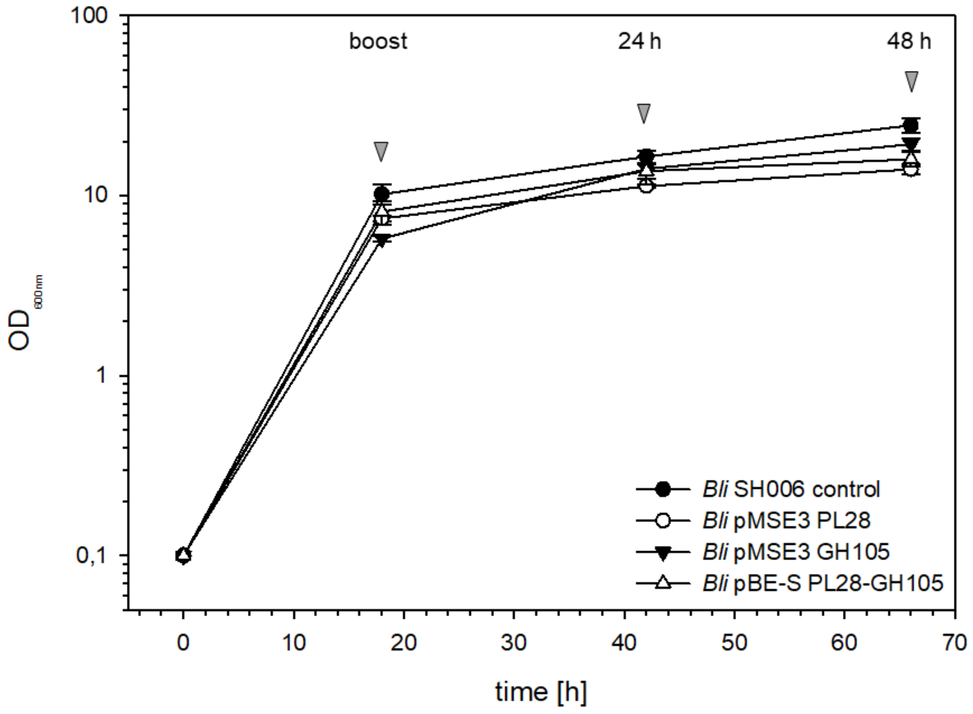

Fig. S11 Growth of the different *B. licheniformis* expression strains. *B. licheniformis* SH006, *B. licheniformis* pMSE3 PL28, *B. licheniformis* pMSE3 GH105 and *B. licheniformis* pBE-S PL28-GH105 were grown under simulated fed-batch conditions in Expresso-B medium at 30°C and 250 rpm. Sampling points for activity measurements, 24 h and 48 h after the boost are indicated.

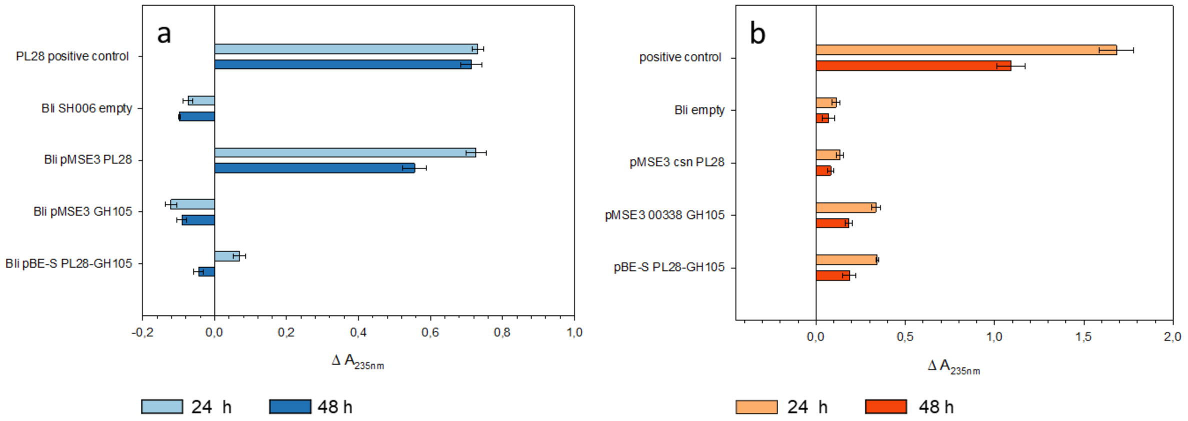

Fig. S12 Activity assay results from *B. licheniformis* SH006, PL28 and GH105 single- and co-expression strain. (**a**) Results of the ulvan lyase (PL28) assay measurement of the lyase product formation at 235 nm over 60 min. The deviation of the absorption of the end-start shows the lyase product formation (lyase activity). (**b**) The deviation of the absorption start-end shows the reversed reaction of the GH105 activity by cleaving of the lyase moiety using prehydrolyzed ulvan from the recombinantly expressed ulvan lyase PL28. Corresponding C-PAGE gels are shown in Fig. S13.

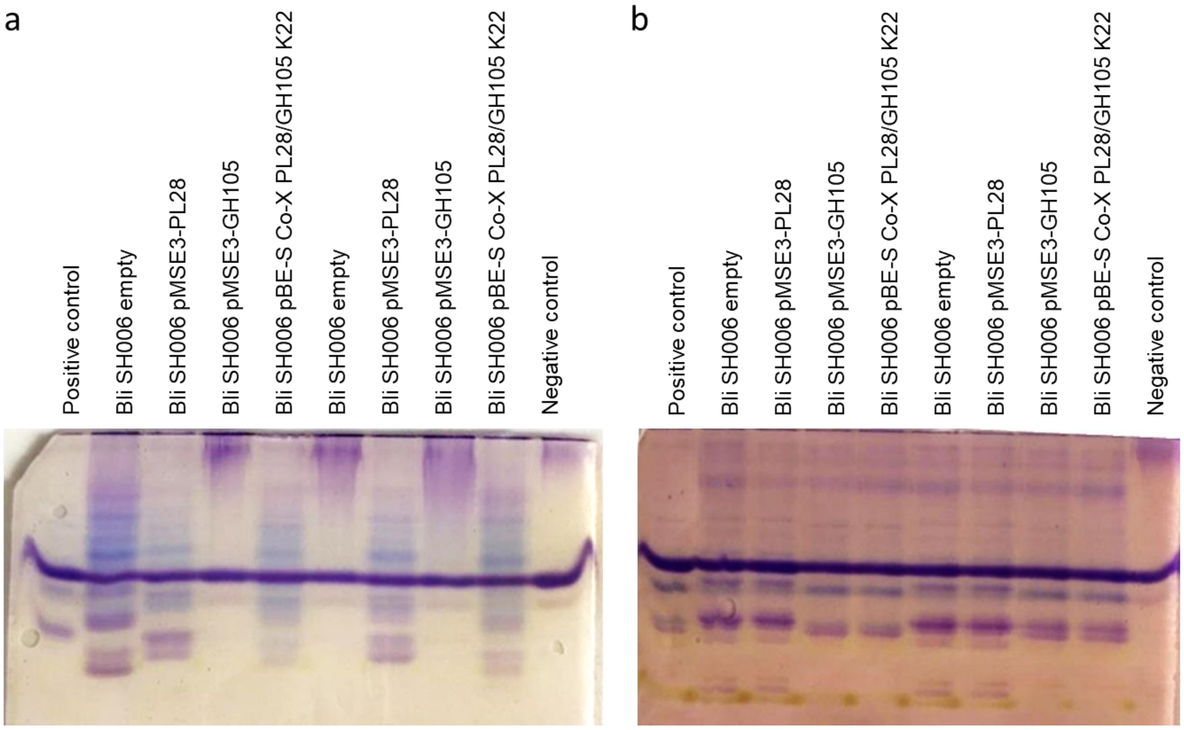

Fig. S13 C-PAGE results from *B. licheniformis* SH006 PL28, GH105 and co-expression strains. The results correspond to the ulvan lyase assay shown in Fig. S12. (**a**) Shows the C-PAGE result of the PL28 ulvan lyase activity (extracellular fraction) of the strain constructs *B. licheniformis* SH006 (Bli SH006) empty, pMSE3_PL28, pMSE3- GH105 or pBE-S Co-X PL28/GH105 K22 (**b**) shows the reaction of the GH105 cleaving of the lyase moiety using prehydrolyzed ulvan.

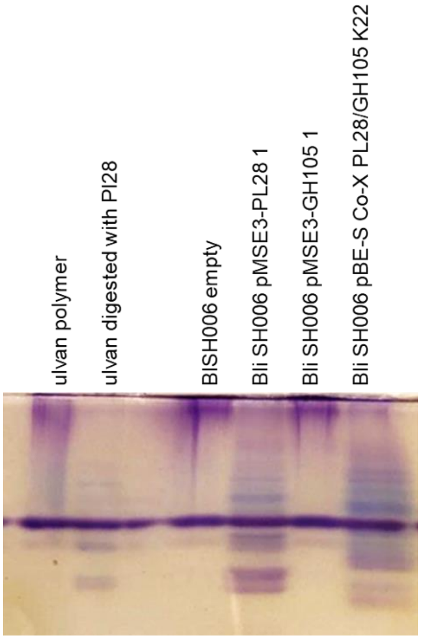

Fig. S14 C-PAGE from the cultivation supernatant of *B. licheniformis* strains in M9-mineral media. *B. licheniformis* (Bli) SH006 PL28, GH105 and coexpression strains were cultivated in M9-mineral media (see Fig. 6) for seven days. A supernatant sample was loaded, as negative control the ulvan polymer and as a positive control the predigested ulvan with PL28 was loaded on the gel.

References

1. Reisky L, Précoux A, Zühlke MK, Bäumgen M, Robb CS, Gerlach N, et al. A complex enzyme cascade degrades the polysaccharide ulvan from green algae. Nat Chem Biol. 2019;803–12.

2. Veith B, Herzberg C, Steckel S, Feesche J, Maurer KH, Ehrenreich P, et al. The complete genome sequence of *Bacillus licheniformis* DSM13, an organism with great industrial potential. J Mol Microbiol Biotechnol. 2004;7:204–11.

3. Hofmeyer T, Hackenschmidt S, Nadler F, Thürmer A, Daniel R, Kabisch J. Draft genome sequence of *Cutaneotrichosporon curvatus* DSM 101032 (Formerly *Cryptococcus curvatus*), an oleaginous yeast producing polyunsaturated fatty acids. Genome Announc. 2016;4:e00362-16.

4. Pohlmann A, Fricke WF, Reinecke F, Kusian B, Liesegang H, Cramm R, et al. Genome sequence of the bioplastic-producing “Knallgas” bacterium *Ralstonia eutropha* H16. Nat Biotechnol. 2006;24:1257–62.

5. Waschkau B, Waldeck J, Wieland S, Eichstädt R, Meinhardt F. Generation of readily transformable *Bacillus licheniformis* mutants. Appl Microbiol Biotechnol. 2008;78:181–8.

6. Yin Y, Mao X, Yang J, Chen X, Mao F, Xu Y. dbCAN: a web resource for automated carbohydrate-active enzyme annotation. Nucleic Acids Res. 2012;40:W445–51.

7. Lu D, Wörmann ME, Zhang X, Schneewind O, Gründling A, Freemont PS. Structure-based mechanism of lipoteichoic acid synthesis by *Staphylococcus aureus* LtaS. Proc Natl Acad Sci. Proceedings of the National Academy of Sciences; 2009;106:1584–9.

8. Stülke J, Hillen W. Regulation of Carbon Catabolism in *Bacillus* Species. Annu Rev Microbiol. 2000;54:849–80.
